# A generalized framework for elliptic curves based PRNG and its utilization in image encryption

**DOI:** 10.1038/s41598-022-17045-x

**Published:** 2022-08-02

**Authors:** Sherif H. AbdElHaleem, Salwa K. Abd-El-Hafiz, Ahmed G. Radwan

**Affiliations:** 1 Engineering Mathematics Department, Faculty of Engineering, Cairo University, Giza, 12613 Egypt; 2 School of Engineering and Applied Sciences, Nile University, Giza, 12588 Egypt

**Keywords:** Computational science, Information technology

## Abstract

In the last decade, Elliptic Curves (ECs) have shown their efficacy as a safe fundamental component in encryption systems, mainly when used in Pseudorandom Number Generator (PRNG) design. This paper proposes a framework for designing EC-based PRNG and maps recent PRNG design techniques into the framework, classifying them as iterative and non-iterative. Furthermore, a PRNG is designed based on the framework and verified using the National Institute of Standards and Technology (NIST) statistical test suite. The PRNG is then utilized in an image encryption system where statistical measures, differential attack measures, the NIST statistical test suite, and system key sensitivity analysis are used to demonstrate the system's security. The results are good and promising as compared with other related work.

## Introduction

Rapid developments in the digital world highlighted the need for securing digital content, especially images as they are used and shared extensively. Therefore, securing images has gained much attention from researchers in the last decade. However, methods of securing images vary a lot depending on the application. For example, image encryption obscures the image while image watermarking transparently embeds ownership. A source of randomness exists in the heart of any encryption system; this source provides the system with its strength and can vary from one system to another. Chaos-based, non-chaos-based, and elliptic curves are sources that proved their efficiency.

Chaos-based techniques gained much attention because of their sensitivity to system parameters and initial conditions. While some techniques added extra parameters to chaotic systems to increase their sensitivity and system key length^1–3^, others generated dynamic S-box using either Henon map^4^ or logistic-sine map^5^, or generated random keystream using quantum logistic map^6^. On the other hand, non-chaos-based systems gained attention from the diversity of components that can be combined to achieve comparable security strength. For example, such systems can utilize the complex details of fractals in the PRNG process^7,8^, use Linear Feedback Shift Register (LFSR) in image encryption^9^, perform permutation and substitution using Feistel networks^10^, or apply a DNA encoding process of image pixels^11^. Moreover, two assessment measures were developed for the performance of various chaotic and non-chaotic based permutation techniques^12^. A summary of several encryption system configurations, based on chaotic and non-chaotic generators, was proposed by Ref.^13^ demonstrating the effect of each configuration on system security.

ECs are utilized because of the difficulty of the Discrete Logarithm Problem (DLP) and the ability to achieve high-security strength using a smaller key length than other public-key techniques. For instance, designing an authenticated encryption scheme for message mapping on EC^14,15^, generating discrete chaotic sequences using the EC-based linear congruential method^16^, using isomorphic elliptic curves in generating S-boxes^17^, improving the ElGamal encryption technique by solving data expansion issue^18,19^, or utilizing the Diffie–Hellman key exchange protocol and EC point addition in image encryption^20^ are among the techniques that utilize ECs.

The main contributions of this paper are summarized as follows. First, a novel generalized framework for EC-based iterative and non-iterative PRNG is proposed and verified using recent literature. With the help of this framework, a simple PRNG based on ECs is designed using one EC point addition operation and two truncations. In addition, an image encryption system combining chaos and number theory is designed by utilizing the proposed PRNG. Finally, the PRNG and encryption system are evaluated using well-known standard criteria and they demonstrated good results.

The paper is organized as follows. After briefly describing the mathematical basics of ECs, a novel framework for EC-based PRNG is presented, and a PRNG is proposed based on it. An image encryption system is, then, designed by utilizing the proposed PRNG. Furthermore, the different evaluation criteria are explained and used in assessing the PRNG and the encryption system. Finally, a comparison with related literature is given, followed by the conclusions.

## Elliptic curves basics

A Weierstrass equation takes the form ${y}^{2}={x}^{3}+\mathrm{A}x+\mathrm{B},$ where A and B are constants. An EC is defined over a field $F$ when $\mathrm{A},\mathrm{ B}\in F.$ For the cubic equation not to have multiple roots, a restriction is added over the values of $A$ and $B$, which is $4{A}^{3}+27{B}^{2}\ne 0$^21^.

For cryptography applications, $x$, $y$, $A$, and $B$ are taken to be elements from the finite fields ${F}_{p}$, where $p$ is a large prime. Adding the point at infinity $\mathcal{O}$ to the set of all points satisfying the EC equation creates an additive abelian group with $\mathcal{O}$ being the identity element. Group operations are point addition and multiplication. Let ${P}_{1}=({x}_{1},{y}_{1})$ and ${P}_{2}=({x}_{2},{y}_{2})$ be points on an EC, $E$, then ${{P}_{3}=P}_{1}+{P}_{2}$ = $({x}_{3},{y}_{3})$ is calculated using

1 $${P}_{3}=\left\{\begin{array}{ccc}\left({m}^{2}-{x}_{1}-{x}_{2},m\left({x}_{1}-{x}_{3}\right)-{y}_{1}\right),& m=\frac{{y}_{2}-{y}_{1}}{{x}_{2}-{x}_{1}},& {x}_{1}\ne {x}_{2}\\ \left({m}^{2}-2{x}_{1},m\left({x}_{1}-{x}_{3}\right)-{y}_{1}\right),& m=\frac{3{{x}_{1}}^{2}+A}{2{y}_{1}},& {P}_{1}={P}_{2}, {y}_{1}\ne 0\\ \mathcal{O},& & {x}_{1}={x}_{2}, {y}_{1}\ne {y}_{2}\\ \mathcal{O},& & {P}_{1}={P}_{2}, {y}_{1}=0\\ {P}_{1},& & {P}_{2}=\mathcal{O}\\ {P}_{2},& & {P}_{1}=\mathcal{O}\end{array}\right.$$

**Figure 1:**
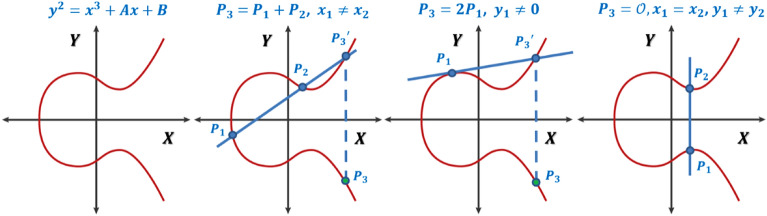
Example EC and the first three cases for point addition.

The geometrical interpretation for the first three cases of point addition on EC is summarized in Fig. 1. Point multiplication by a value $n$ is treated as successively adding the point to itself $n$ times. An efficient implementation for point multiplication is the point doubling algorithm^21^.

The order of a point $P$ is the smallest positive integer $k$ such that $kP=\mathcal{O}$. The order of a point $P$ always divides the order of the group $E\left({F}_{p}\right)$. Let $G$ be a point on the EC,$E$, then $G$ is called a generator point with order $N$ for the cyclic subgroup consisting of the points $\{G,2G,3G,\dots , NG= \mathcal{O}\}$.

## Framework for EC-based PRNGs

A PRNG is a critical element in any encryption system as it provides the system with a pseudorandom keystream. A good design of a PRNG should be sensitive to the initial state, give uniform distribution of output bits, and the period should be large enough to resist cryptanalysis attacks^22^.

**Figure 2:**
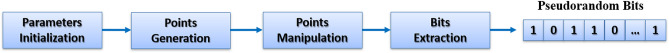
A generalized framework for PRNGs.

EC points are the primary source for any EC-based PRNG, which can generally fall into two schemes. The first scheme picks a generator point with a large order group and applies group operations to calculate new points and extract the random bits from the coordinates of each point. On the other hand, the second scheme calculates all required EC points, and then the coordinates of the points are used in producing the random bits. In this sense, a framework can be established where both design schemes can fit in. While the first scheme is called iterative because the points are generated one at a time, the second scheme is called non-iterative since all points are generated simultaneously. The proposed framework, shown in Fig. 2, consists of the following four main blocks.

- Parameters initialization: In this stage, EC parameters are initialized. In some design cases, other systems are integrated into the process and, hence, those system parameters are also initialized in this stage. For example, suppose that a chaos-based system is integrated into the design to enhance the randomness of the process and add extra complexity against different attacks. In this case, all parameters required by this chaotic system are initialized.
- Points generation: In the case of iterative designs, only one point is generated per iteration using an iterative equation. In general, the iterative equation consists of group operations such as point addition, doubling, and multiplication. The more operations exist, the more complex the generator is. In the case of non-iterative designs, all points required by the generator are calculated by evaluating the EC equation for all possible values of $x$ or randomly selected values of $x$ using some criteria.
- Points manipulation: In this stage, the produced points are processed based on some design criteria. For instance, the coordinates of the points can be converted into binary form. Other designs can use the coordinates values and apply mathematical formulas to produce a number.
- Bits extraction: This stage processes the output from the previous stage and generates the required pseudorandom bits. For example, a common logic in this stage includes bit truncation to satisfy particular design criteria.

Table 1 compares iterative and non-iterative designs with respect to different aspects. Clearly, each design category has its advantages. Depending on the application, the designer should choose the design that is more suitable. For instance, in applications that work with unknown data lengths like voice calls, it is better to use an iterative design as the period of the PRNG will be long enough to cover the amount of data that needs to be encrypted. In applications that work with known data length, like images, non-iterative designs can pick an EC with enough points to achieve the required period for PRNG. In the following subsections, some recent EC-based PRNG literatures are discussed and mapped into the proposed framework, which demonstrates the framework's flexibility.

**Table 1:** Comparing iterative and non-iterative designs.

	Iterative design	Non-iterative design
EC selection	Predefined secure ECs/randomly generated ECs	Random generation of ECs
EC prime $p$	Very large (in the order of 192 bits or more)	Small (in the order of 16 bits or less)
EC points	Iteration over points of a cyclic subgroup	Evaluating the EC equation for all possible values of $x$
Period	Usually around the order of the generator point	It depends on the number of points generated
Suggested applications	Unknown or known data length (e.g., voice calls and video streaming/images)	Known data length (e.g., images and data files)

### Iterative designs

**Figure 3:**
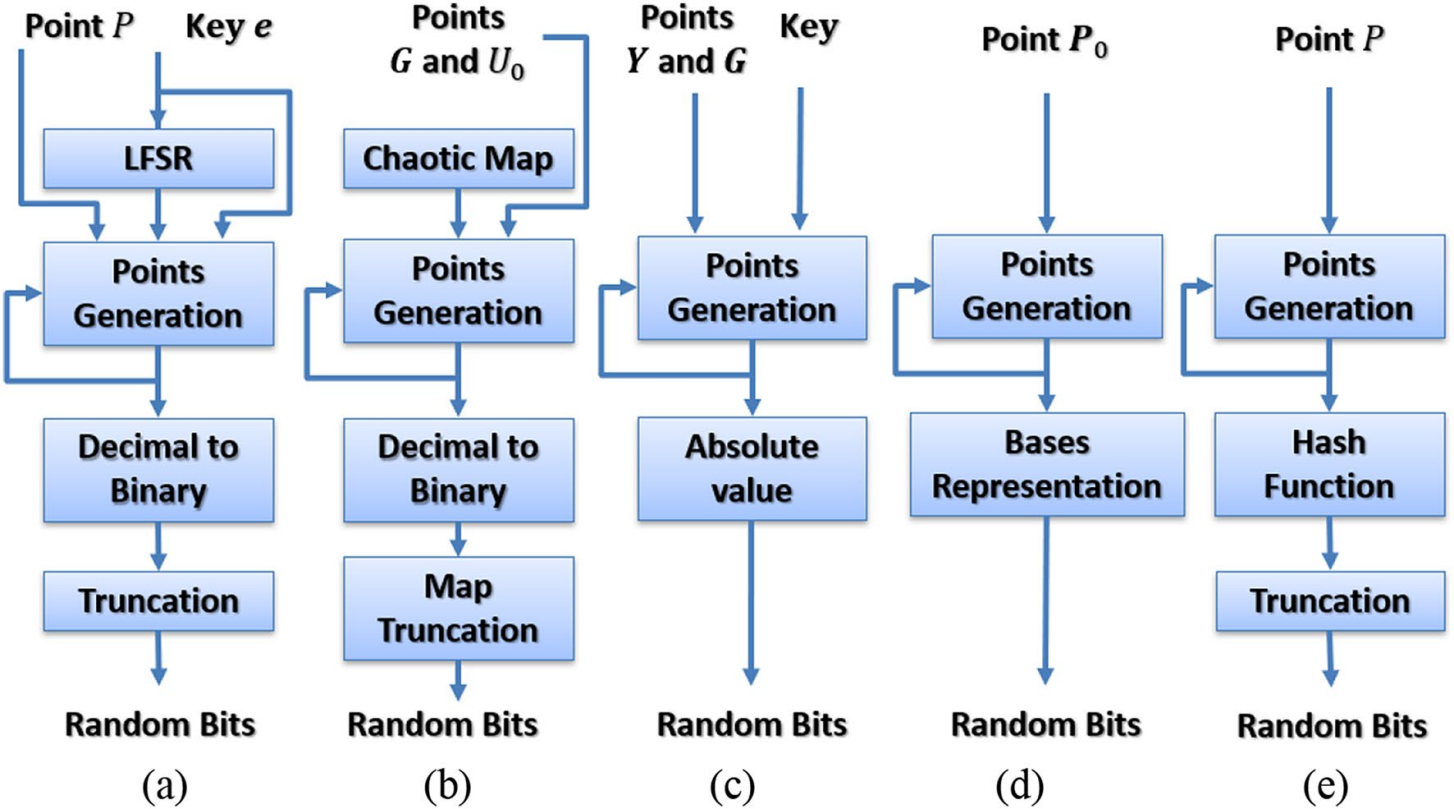
Simplified block diagrams for the iterative techniques in (**a**) Ref.^23^, (**b**) Ref.^24^, (**c**) Ref.^25^, (**d**) Ref.^26^, and (**e**) Ref.^27^.

Several iterative PRNG algorithms were introduced during the last decade, such as the techniques shown in Fig. 3, where a simplified block diagram for each technique is depicted. Table 2 demonstrates the mappings of those techniques into the proposed framework.

**Table 2:** Mapping of the surveyed iterative techniques into the proposed framework.

Ref. no.	Parameters initialization	Points generation	Points manipulation	Bits extraction	Notes
Ref.^23^, 2015	Point *P* on the curve and a key $e$ Using $e$, find ${K}_{0}$ Using $e$, initialize LFSR	LFSR outputs ${C}_{i}$ ${K}_{i}=X({K}_{i-1}P)+{C}_{i-1}$ ${S}_{i}={K}_{i}P+{K}_{0}P$	Convert the x-coordinate of ${S}_{i}$ to binary form	Apply truncation on x-coordinate bits	The LFSR increased the period and introduced randomness in the keystream
Ref.^24^, 2015	Pick an EC, $E$, and a generator point $G$ on E Point ${U}_{0}\in E({F}_{p})$	Increment index $i$ Use the chaotic map to get the binary sequence ${b}_{i}$ ${U}_{i}=i\left(1+{b}_{i}\right)G+{U}_{0}$	Convert the point ${U}_{i}$ into its binary form	Apply the map ${U}_{2\times 2}(x,y)$ or the map ${U}_{3\times 3}(x,y)$ on the point ${U}_{i}$, where ${U}_{\mathrm{k}\times \mathrm{k}}$ takes the rightmost $k$ bits from $x$ and $y$ coordinates	Different chaotic maps can be used The chaotic map increased the randomness of the bitstream
Ref.^25^, 2017	Two points $Y$ and $G$ ${SK}_{1}=$ primary key	$A= {SK}_{i}G$ $B=A+Y$ $C=B+G$ ${SK}_{i+1}={y}_{A}+{y}_{B}+{y}_{C}$	${Z}_{i}=\left|{x}_{A}\times {x}_{B}\times {x}_{C}\right|$	Read the value ${Z}_{i}$	The two points $Y$ and $G$ have very high orders
Ref.^26^, 2019	Point ${P}_{0}$ of order $n$ Pick $r\in [1,n-1]$ let ${\alpha }_{1}, \dots ,{\alpha }_{p}$ be a basis of ${F}_{{2}^{p}}$	${P}_{k}={r}^{k}{P}_{0}$ ${x}_{k}=X({P}_{k})$	Writing ${x}_{k}$ =${{s}_{k}}^{(1)}{\alpha }_{1}+\dots +{{s}_{k}}^{(p)}{\alpha }_{p}$	Read the sequence ${{s}_{k}}^{(i)} , i=1, \dots , p$	$n$ has a large prime order $r$ has a large multiplicative order mod *n*
Ref.^27^, 2020	Select secure EC Select point $P$ ${S}_{0}=X(P)$	$\varphi$ is a truncation function $H$ is a hash function ${S}_{i}={\varphi (x[S}_{i-1}P])$ ${h}_{i}=\varphi (H\left({S}_{i}\right))$	Apply $\varphi$ on the x-coordinate of ${S}_{i-1}P$ Apply $\varphi$ on $H\left({S}_{i}\right)$	Read lower-order bits from ${h}_{i}$	The hash function enhanced the statistical properties of the output bits

### Non-iterative designs

**Figure 4:**
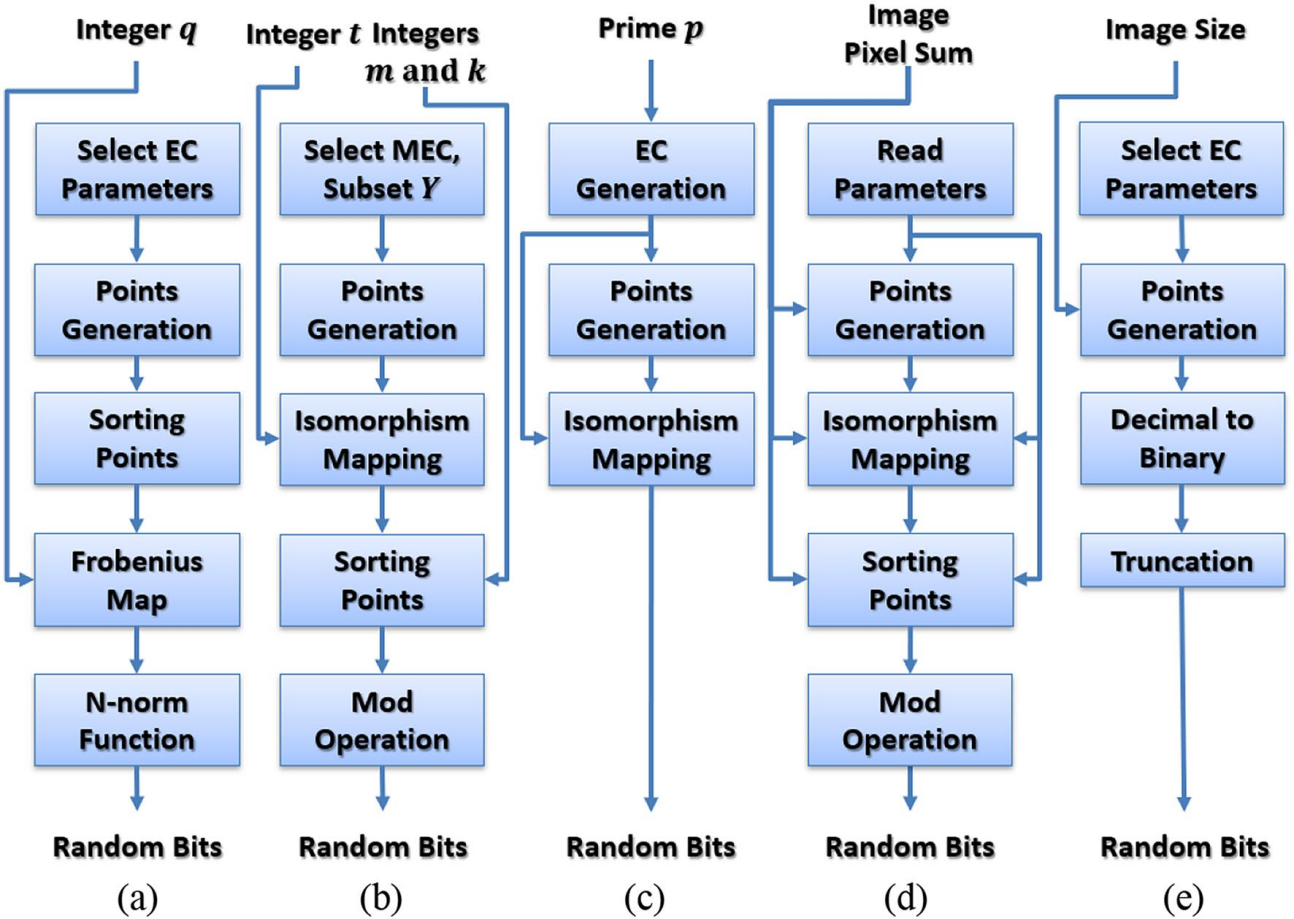
Simplified block diagrams for the non-iterative techniques in (**a**) Ref.^28^, (**b**) Ref.^29^, (**c**) Ref.^30^, (**d**) Ref.^31^, and (**e**) Ref.^32^.

Several non-iterative PRNG designs were proposed during the last decade, such as the designs shown in Fig. 4, where a simplified block diagram for each design is depicted. Table 3 demonstrates the mappings of those designs into the proposed framework.

**Table 3:** Mapping of the surveyed non-iterative techniques into the proposed framework.

Ref. no.	Parameters initialization	Points generation	Points manipulation	Bits extraction	Notes
Ref.^28^, 2019	Randomly select EC parameters ($p,a,b$) Pick $q$ as a parameter for Frobenius map	Apply brute force search on EC	Sorting points Apply Frobenius map on points Apply n-norm on projected points, then approximate to the nearest integer	Read integers after approximation	Azam et al.^33^ introduced the ordering of EC points used to sort the points of EC
Ref.^29^, 2021	Select a Mordell Elliptic Curve (MEC) $E$ Select a subset $Y\subseteq [0,p-1]$ Select two integers $m$ and $k$ Select $t\in [1,\frac{p-1}{2}]$ Select a total order operator ${<}^{*}$	For each integer $y$ in $Y$ find the point $\left(x,y\right)$ Calculate the point $\left({t}^{2}x,{t}^{3}y\right)$ then add it to set $A$	Sort the set $A$ using the total order operator ${<}^{*}$	Read the y-coordinate $mod m$ from the sorted list	MEC has the property of $a = 0$ $p\equiv 2 \; mod \; 3$
Ref.^30^, 2021	Select large prime $P$ Generate the curve ${E}_{a}^{P}$ using brute force technique	Apply brute force search on ${E}_{a}^{P}$	${\varphi }_{\gamma }(u,v)=\frac{v+\gamma u}{v-\gamma u}$ ${\gamma }^{2}=a$ Use isomorphism ${\varphi }_{\gamma }$ to map all points of ${E}_{a}^{P}$ to ${F}_{p}$	Read mapped integers	${E}_{a}^{P}: {y}^{2}={x}^{3}+\mathrm{a}x$ $\gamma \in {F}_{p}$
Ref.^31^, 2021	Read input parameters Calculate ${S}_{I}$ from plain text	Calculate isomorphic parameter ${t}_{r}$ Map points of ${E}_{p,b}$ to ${E}_{p,{t}_{r}^{6}b}$ using isomorphic parameter ${t}_{r}$	Select ordering ${O}_{r}$ Select subset $A\subset {E}_{p,{t}_{r}^{6}b}$ Sort $A$ using ${O}_{r}$ Pick a subset ${A}_{r}\subseteq [0,p-1]$ Select an integer ${h}_{r}$ Sort ${A}_{r}$ using ordering ${<}^{*}$ which depend on $A, {O}_{r}$ and ${h}_{r}$	Calculate ${m}_{r}$ Apply $mod {m}_{r}$ to elements of ${A}_{r}$ Read reduced elements of ${A}_{r}$	The PRNG is based on MECs The PRNG output is very sensitive to plain text
Ref.^32^, 2022	Read EC secp256r1 parameters Read image size	For all pixels in the image, generate random points from the curve	Convert the y-coordinate of each point to binary form	Read the least significant 8 bits from each y-coordinate	The random generation of points is based on a predefined function

In summary, EC point coordinates, in their binary form, can serve as a good source for random bits. The surveyed literature can be grouped into two categories, iterative and non-iterative. The main disadvantage of the first category is that the iterative equation can include too many EC group operations and may be combined with other operations regarding non-EC elements, which can be complex in limited resource systems. The main disadvantage of the second category is that it cannot be used with large prime numbers, where safe recommended ECs exist, because it is not possible to calculate all curve points. Therefore, this paper proposes to design an iterative PRNG with only one addition operation, which makes it suitable in a limited resource system and can be used in real-time applications using NIST-recommended safe ECs.

## Proposed PRNG

With the proper choice of the EC parameters and a generator point $G$ with a high order, usually a large prime number, the cyclic subgroup generated by the point $G$ can be iterated. Moreover, using each point coordinate, pseudorandom numbers can be extracted. In this paper, a simple PRNG is designed and used in image encryption.

**Figure 5:**
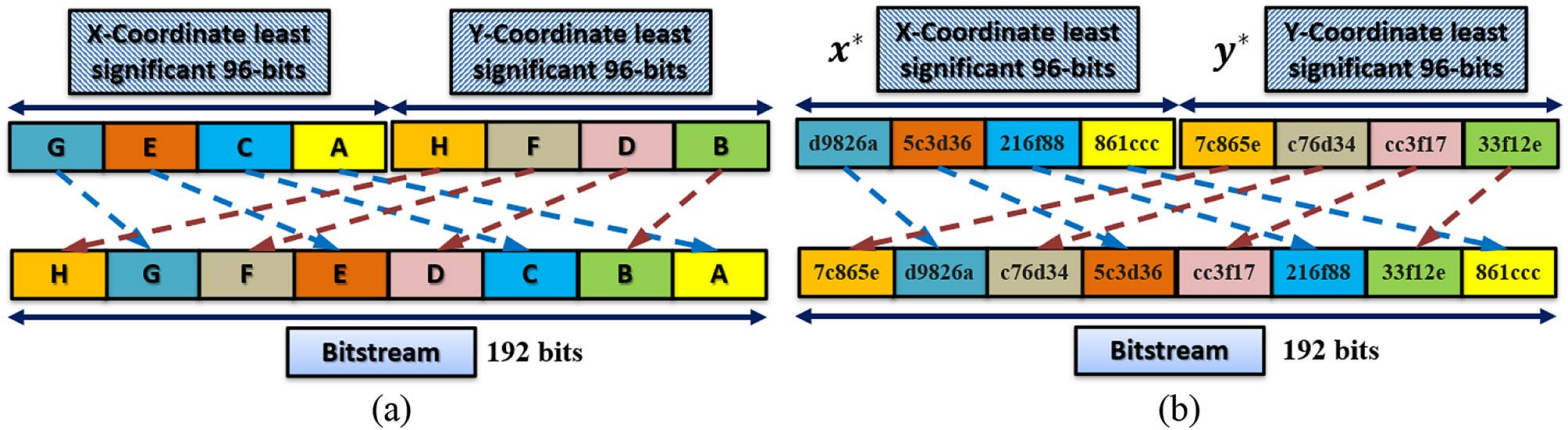
(**a**) Conversion from a point on EC to bitstream representation and (**b**) an example.

The PRNG is based on the iterative equation

2 $${P}_{n+1}={P}_{n}+{P}_{0},$$

where ${P}_{0}=KG$ is the initial base point of the PRNG,$K$ is the system key value, and $G$ is the generator point. If ${P}_{0}$ is changed, a completely new sequence of points is generated. For each point, the $x$ and $y$ coordinates are converted into their equivalent binary representation. Then, the least significant 96 bits from each coordinate are mixed to create a stream of 192 bits, as shown in Fig. 5a. For the example shown in Fig. 5b, consider a point $P(x,y)$. The least significant 96-bits, ${x}^{*}$ and ${y}^{*}$, are extracted from each coordinate, respectively. Then, each 24-bits from ${x}^{*}$ and ${y}^{*}$ are extracted and mixed to form the final bitstream. The resulting bits are random because the hopping from one point to another gives an entirely different point regarding coordinate values, and because of the mixing between the $x$ and $y$ coordinates. It is important not to extract more bits from each coordinate because higher bits are not chaotic enough, and the more bits used, the more the bitstream is not secure and can be attacked as pointed out in Ref.^34^.

In the PRNG design, every point from the EC can produce 192 bits, and since the generator is used to encrypt images, every 24 bits (no. of bits in each pixel) are parsed from the bitstream and then used to encrypt the image pixel. Hence, in pixel terms, a total of $192/24=8$ pixels can be encrypted using only one point from the EC.

**Figure 6:**
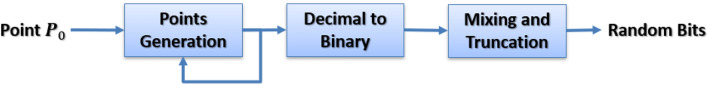
Simplified block diagram for the proposed PRNG**.**

The PRNG design is inspired by the proposed framework, where the number of operations in each stage is minimized to achieve better performance. Figure 6 shows the simplified block diagram for the proposed PRNG, whereas Table 4 shows the mapping of this design into the proposed framework. The proposed PRNG has only one EC addition operation in the points generation stage, which helps in speeding up the time consumed in this stage. Furthermore, only decimal to binary conversion is applied in the points manipulation stage, and mixing (bit shifting) and truncation operations are performed in the bits extraction stage. In this sense, the design of the PRNG is optimized for speed and low resources.

**Table 4:** Mapping of the proposed PRNG into the proposed framework.

Parameters initialization	Points generation	Points manipulation	Bits extraction	Notes
Select secure EC Select $K$ ${P}_{0}=KG$	Increment index $n$ ${P}_{n+1}={P}_{n}+{P}_{0}$	Convert the $x$ and $y$ coordinates of the point ${P}_{n}$ into its binary form	Read least 96 bits from both $x$ and $y$ coordinates Mix the bits from $x$ and $y$ coordinates	Any secure curve can be used $K$ is at least 128 bits

In practice, the EC parameters and $G$ should be chosen such that the order of $G$ is a large prime number. Hence, the period of such PRNG is significantly large enough to be used in encryption applications. In this work, the PRNG uses Curve-192, although any other recommended secure curve can be used as well. This curve is one of the NIST's recommended curves^35^; its prime modulus $p$ is 192 bits, the base point $G$ has 189 bits and 187 bits in the $x$ and $y$ coordinates, respectively, and its order $n$ is 192 bits. Iterating the cyclic group generated by $G$, the average number of bits in each point $x$ and $y$ coordinates is close to that of the generator point $G$.

## Proposed encryption system

**Figure 7:**
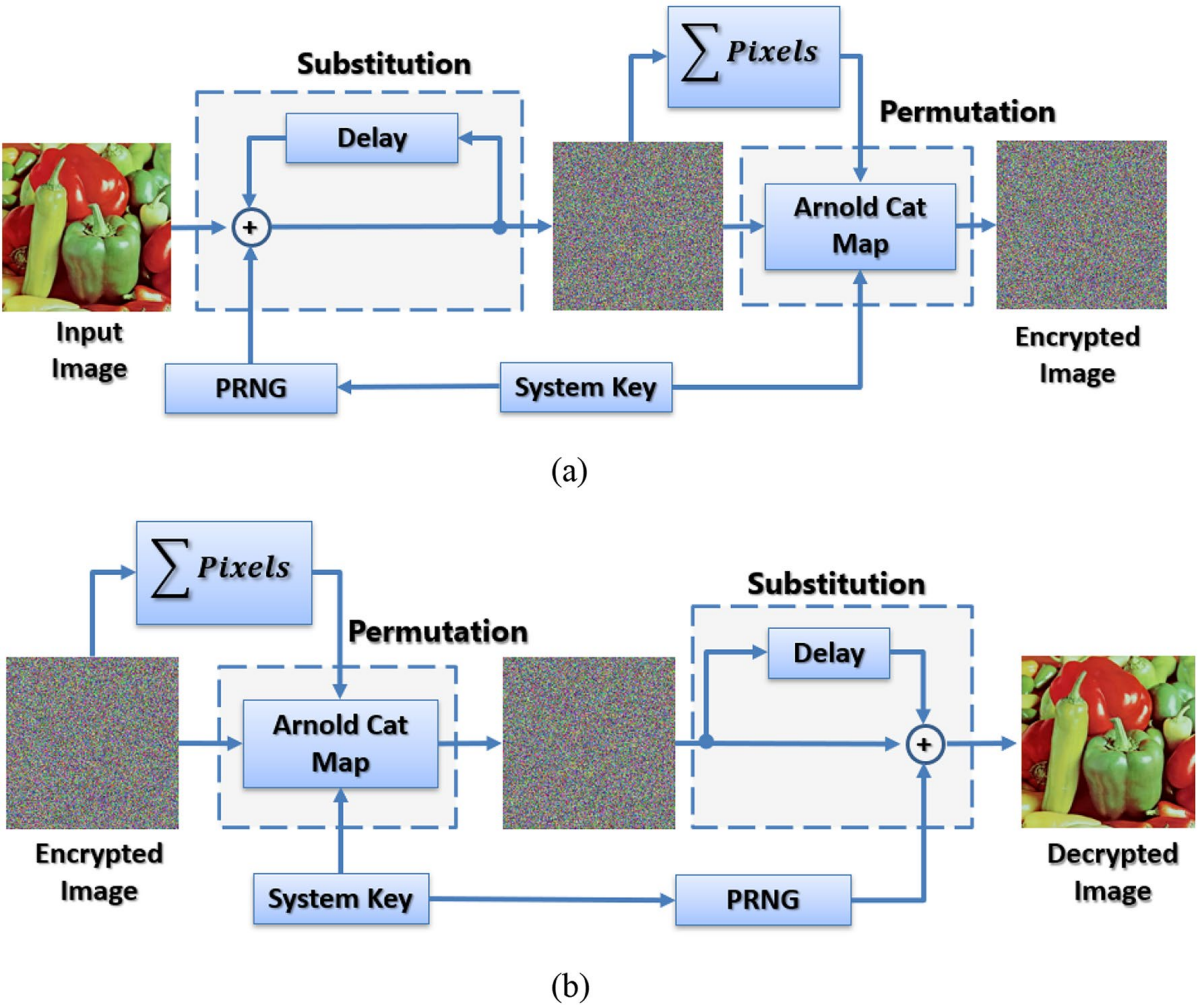
Block diagram for (**a**) the encryption system and (**b**) the decryption system.

The block diagram of the proposed encryption system is shown in Fig. 7, where the system consists of two main stages necessary to achieve Shannon's confusion and diffusion properties^36^. The first stage is the substitution stage, where pixel values are changed. The second stage is the permutation stage, where pixel locations are shuffled across the image. For the system to be sensitive to input changes, the algebraic sum of all pixels in the three channels is calculated and used to modify the permutation stage parameters. In this sense, the system is protected from different differential attack attempts.

### Substitution stage

In this stage, the output from the PRNG is $Xored$ with the image pixel. In addition, a delay element is used to make the current encrypted pixel's value dependent on the last encrypted pixel value. Hence, this provides the system with more strength against differential attacks.

The substitution phase can be represented using the equation

3a $${E}_{R} = {RN}_{i}   \oplus  {I}_{R}   \oplus   { D}_{R},$$

3b $${E}_{G} = {RN}_{i+1}   \oplus   {I}_{G}   \oplus   {D}_{G},$$

3c $${E}_{B} = {RN}_{i+2}   \oplus   {I}_{B}   \oplus   {D}_{B},$$

where ${E}_{R}$, ${E}_{G}$, and ${E}_{B}$ are the encrypted pixel values for the red, green, and blue channels, respectively.${RN}_{i}$ is the *i*th byte from the PRNG bitstream. ${I}_{R}$, ${I}_{G}$, and ${I}_{B}$ are the image pixel values for the red, green, and blue channels, respectively. ${D}_{R}$, ${D}_{G}$, and ${D}_{B}$ are the previous encrypted pixel values for the red, green, and blue channels, respectively, and each is initialized with the value of 0.

### Permutation stage

**Figure 8:**
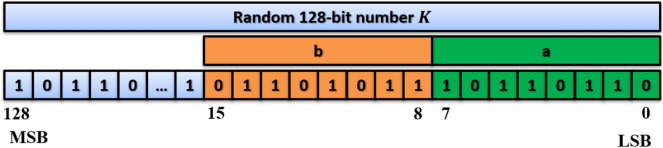
System key construction.

Arnold's cat map is used in permuting the image pixels, as defined by:

4 $$\left(\genfrac{}{}{0pt}{}{{x}_{new}}{{y}_{new}}\right)= \left(\begin{array}{cc}1& a\\ b& 1+ab\end{array}\right)\left(\genfrac{}{}{0pt}{}{x}{y}\right)modM,$$

where $a,b \in \left\{ 1, 2,\dots , M-1\right\}, M$ is the square matrix size,$x,y \in \left\{1, 2,\dots , M\right\}$ are the original pixel location and ${x}_{new},{y}_{new}$ are the new pixel location. The values of $a$ and $b$ are calculated from the system key and then modified using:

5a $$S=sum\left(image \; pixels\right),$$

5b $$a=mod\left(S+{a}_{key},M-1\right)+1,$$

5c $$b=mod\left(S+{b}_{key},M-1\right)+1,$$

where ${a}_{key},{b}_{key}$ are 8-bit numbers extracted from the system key as shown in Fig. 8, and $mod$ returns the remainder after division.

### System key

The system key should be at least 128 bits, long enough to resist brute-force attacks in cryptographic applications. Furthermore, any change in the key, even a one-bit change, should produce completely different output from the original key. As shown in Fig. 8, a random 128-bit number $K$ is selected to be the system key where Arnold's cat map parameters $a$ and $b$ are extracted from this key.

For security purposes, the generator point $G$ provided by the NIST Curve-192 cannot be used as the base point of the PRNG. Therefore, in the beginning, the point ${P}_{0}=KG$ is calculated. It is worth mentioning that the large value of $K$ will not affect the speed of calculating the point ${P}_{0}$ as mentioned earlier in the introduction.

## Evaluation criteria

This section discusses different evaluation criteria used to evaluate the proposed PRNG and encryption system.

### NIST statistical test suite

NIST SP-800-22 is a group of 15 tests applied on bitstreams to decide the randomness of the bits^37^. If any of the tests failed, the bitstream is not recommended to be used in cryptography applications. The output from this test is validated by the P-value distribution (PV) and the proportion of passing sequences (PP). For a truly random sequence, the PV is equal to 1, while for a nonrandom sequence, the PV approaches 0. A significant value $\alpha$ controls the success of each test. If PV $\ge \alpha$, then the sequence passes the test, otherwise, it fails the test. In case of cryptography applications, $\alpha =0.01$, which means that if more than 1% of the sequence fails the test, then the complete sequence is considered nonrandom.

### Correlation coefficients of image pixels

This metric measures how much image pixels are correlated to each other. This measure is generally applied to adjacent pixels in the horizontal, vertical, and diagonal directions. It is calculated using:

6a $$Cov\left(x,y\right)=\frac{1}{\mathrm{N}}\sum_{\mathrm{i}=1}^{\mathrm{N}}\left({\mathrm{x}}_{\mathrm{i}}-\frac{1}{\mathrm{N}}{\sum }_{\mathrm{j}=1}^{\mathrm{N}}{\mathrm{x}}_{\mathrm{j}}\right)\left({\mathrm{y}}_{\mathrm{i}}-\frac{1}{\mathrm{N}}{\sum }_{\mathrm{j}=1}^{\mathrm{N}}{\mathrm{y}}_{\mathrm{j}}\right),$$

6b $$D\left(x\right)=\frac{1}{\mathrm{N}}{\sum }_{\mathrm{i}=2}^{\mathrm{N}}{\left({\mathrm{x}}_{\mathrm{i}}-\frac{1}{\mathrm{N}}{\sum }_{\mathrm{j}=1}^{\mathrm{N}}{\mathrm{x}}_{\mathrm{j}}\right)}^{2},$$

6c $$\rho = \frac{Cov\left(x,y\right)}{\sqrt{\mathrm{D}\left(\mathrm{x}\right)}\sqrt{\mathrm{D}\left(\mathrm{y}\right)}},$$

where $\mathrm{N}$ is the number of elements in the two vectors $x$ and $y$. For typical images, the value of $\rho$ is close to 1, while for encrypted images, the value of $\rho$ should be closer to 0.

### Differential attack measures

This attack studies the relationship between two encrypted images after changing one pixel in the source image. Three measures are used, which are the Mean Absolute Error (MAE), the Number of Pixels Change Rate (NPCR), and the Unified Average Changing Intensity (UACI)^38^. Expected values for MAE, NPCR, and UACI are around 100, 99.6%, and 33.34%, respectively. Let $E$ be the source image, $E1$ be the encrypted image and $E2$ be the encrypted image after changing one pixel in the original image, then

7a $$\mathrm{MAE }= \frac{1}{W\times H}\sum_{\mathrm{i}=1}^{H}\sum_{\mathrm{j}=1}^{W}|E1\left(\mathrm{i},\mathrm{j}\right)-E\left(\mathrm{i},\mathrm{j}\right)|,$$

7b $$D\left(i,j\right)=\left\{\begin{array}{c}\begin{array}{cc}0 & E1(i,j) = E2(i,j)\end{array}\\ \begin{array}{cc}1& E1(i,j) \ne E2(i,j)\end{array}\end{array}\right.,$$

7c $$NPCR=\frac{1}{W \times H}\sum_{i=1}^{H}\sum_{j=1}^{W}D\left(i,j\right)\times 100\mathrm{ \% },$$

7d $$UACI= \frac{1}{W \times H}\sum_{i=1}^{H}\sum_{j=1}^{W}\frac{|E1\left(i,j\right)- E2\left(i,j\right)|}{255}\times 100\mathrm{ \%},$$

where $W$ and $H$ are the width and height of the image, respectively.

### Mean square error (MSE)

This metric is used to measure the error between two images. Let $E$ be the source image and $D$ be the wrong decrypted image, then

8 $$MSE=\frac{1}{W \times H}\sum_{i=1}^{H}\sum_{j=1}^{W}{\left[E\left(i,j\right)-D(i,j)\right]}^{2}.$$

### Entropy analysis

Entropy is a measure of the predictability of random sources. For a source that produces $N$ symbols with probabilities $P\left({S}_{i}\right), i=1, 2, ..,N$, the entropy of that source is calculated using:

9 $$Entropy= -\sum_{i=1}^{N}P\left({S}_{i}\right){log}_{2}P\left({S}_{i}\right).$$

For a random source, this value approaches ${\mathrm{log}}_{2}N$. In the case of color images, this value approaches 8 for each channel.

## Analysis results

In this section, the randomness and efficiency of the PRNG are, first, demonstrated. Then, the encryption system is evaluated using the Peppers image of size $256\times 256$ as well as some additional images from the USC-SIPI database^39^ of size $512\times 512$. The system key sensitivity is examined by changing one bit and observing the results. Finally, the computation complexity is analyzed and comparisons with related literature are given.

### PRNG results

The proposed PRNG is evaluated using thirty different 128-bit random $K$ values (${K}_{1}, ..., { K}_{30}$). Let ${K}_{1}= ede8a3004ce2b2579c937b3874aba2de$ be a 128-bit random number and let ${K}_{1}^{*}= {K}_{1}+1$. Let ${K}_{2} = fe23c064b1cc841a0027ad705ac47d98$ and ${K}_{3}= b6c575a9a76716fcbccdcf16740fb22b$. The choice of ${K}_{1}^{*}$ was made to test the sensitivity of the PRNG for only a one-bit change in the key. In order to test the PRNG using the NIST test suite, a total of $25165824=24\times {2}^{20}$ bits are generated, equal to the number of bits found in a color image of size $1024\times 1024$.

**Figure 9:**
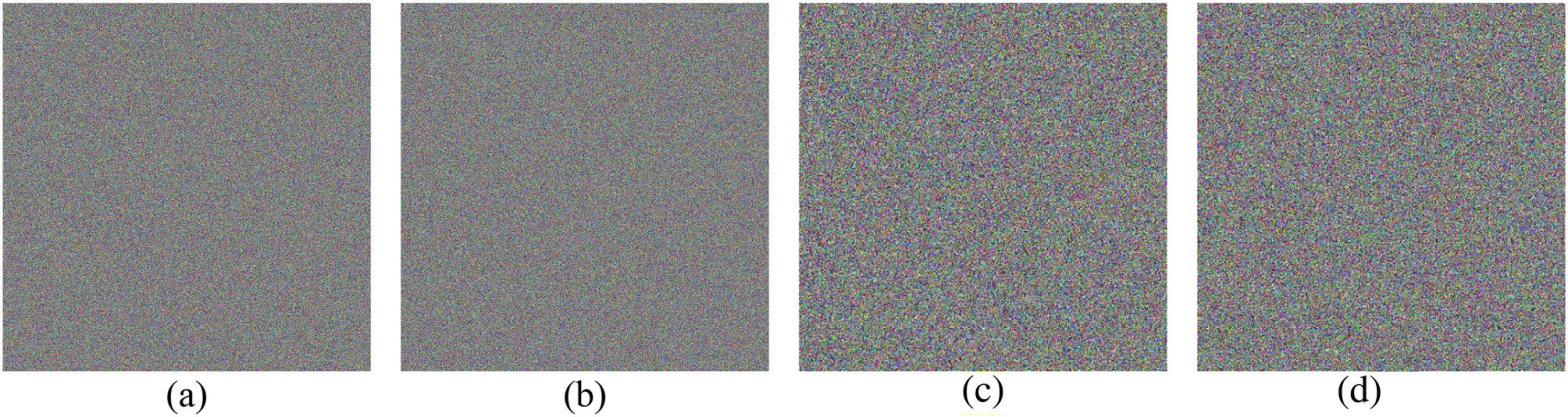
Output bitstreams of the PRNG represented as images in four cases: (**a**) key ${K}_{1}$, (**b**) key ${K}_{1}^{*}$, (**c**) key ${K}_{2}$, and (**d**) key ${K}_{3}$.

The NIST results for the PRNG are shown in Table 5. For the sensitivity test using ${K}_{1}$ and ${K}_{1}^{*}$, the results show that the bitstreams are random and have passed all 15 tests. Furthermore, the bitstreams are converted into two color images, and the results are shown in Fig. 9. Visual inspection of the images supports the NIST results. The correlation between the two bitstreams is calculated and found to be 0.0009, demonstrating the PRNG's sensitivity to one-bit change in the key. As for other test cases, (${K}_{2}, {K}_{3,} \dots , {K}_{30}$), similar results are achieved. Accordingly, Table 5 and Fig. 9 include the results for ${K}_{2}$ and ${K}_{3}$ as representatives for the remaining cases.

**Table 5:** NIST results for the PRNG.

Test	${K}_{1}$		${K}_{1}^{*}= {K}_{1}+1$		${K}_{2}$		${K}_{3}$	
	PV	PP	PV	PP	PV	PP	PV	PP
Frequency	0.637	0.958	0.213	1.000	0.637	1.000	0.437	1.000
Block frequency	0.350	1.000	0.163	1.000	0.637	1.000	0.437	1.000
Cumulative sums	0.592	1.000	0.300	1.000	0.534	1.000	0.508	1.000
Runs	0.276	1.000	0.534	0.958	0.013	1.000	0.437	1.000
Longest run	0.437	1.000	0.740	1.000	0.213	1.000	0.122	1.000
Rank	0.740	0.958	0.740	1.000	0.350	0.958	0.122	1.000
FFT	0.035	0.958	0.637	1.000	0.534	1.000	0.122	1.000
Non-overlapping template	0.339	0.991	0.322	0.993	0.320	0.990	0.345	0.991
Overlapping template	0.834	1.000	0.025	0.958	0.276	0.958	0.013	0.958
Universal	0.163	1.000	0.025	0.958	0.911	1.000	0.437	0.958
Approximate entropy	0.437	1.000	0.740	1.000	0.091	1.000	0.637	1.000
Random excursions	0.055	0.992	0.167	1.000	0.088	0.993	0.311	0.981
Random excursions variant	0.098	1.000	0.218	0.996	0.066	0.964	0.401	0.987
Serial	0.034	0.958	0.437	1.000	0.451	0.979	0.209	1.000
Linear complexity	0.534	1.000	0.834	1.000	0.276	1.000	0.834	0.958
Final result	Success		Success		Success		Success

Table 6 compares some iterative methods with this work. Although all iterative methods can achieve a long period with the proper choice of the EC parameters, the complexity for each technique is not the same. The more operations involved in the design, the more complex the design is. Clearly, the proposed PRNG contains the least number of EC and non-EC operations and, hence, has the least complexity.

**Table 6:** Comparison between iterative methods and this work.

Ref. no.	EC operations	Non-EC operations	EC selection	Period $\mathbf{T}$
Ref.^23^, 2015	Two multiplications One addition	Clocking the LFSR One addition Truncation	ECs defined over the field ${F}_{{2}^{m}}$	$T=C\times {(2}^{m}-1)$ where $C\ge 1$ and $m$ is the length of LFSR in bits
Ref.^24^, 2015	One multiplication One addition	Chaotic map iteration One addition One multiplication	EC defined over ${F}_{p}$	$T<{p}^{1-\delta }$ where $\delta >0$
Ref.^25^, 2017	One multiplication Two additions	Two multiplications Two additions One absolute value	The Internet Engineering Task Force (IETF)^40^	Not given
Ref.^26^, 2019	One multiplication	One power Basis representation	Koblitz EC defined over ${F}_{p}$	$T=(n-1)/2$ where $n$ is the order of generator point
Ref.^27^, 2020	One multiplication	Hash function Truncation	EC defined over ${F}_{p}$	Not given
This Work	One addition	Two truncations	NIST recommended ECs	Order of generator point

The proposed PRNG is examined to determine the bitrate that can be achieved. The experiment is conducted on a Dell laptop with processor Intel Core i7-1065G7 CPU @ 1.30 GHz, running Windows 10 with 16 GB of RAM. Two implementations for the PRNG are considered; the first one uses C# under .net framework 4.7 and the second one uses MATLAB R2015a. The proposed PRNG is run for 30 times, with 65,536 bytes generated in each run. Then, the average bitrate is calculated for both the MATLAB and C# implementations. In the case of MATLAB, the JAVA BigInteger class is used, leading to runtime overhead due to calls between MATLAB and JAVA. In the case of C#, however, no overhead is encountered as C# contains an implementation for the BigInteger class. Table 7 compares the bitrates achieved in Megabits per second (Mbps) by the proposed PRNG and other related PRNGs based on ECs. The bitrates achieved by this work are better than those achieved by other related works, which is attributed to the few used operations as shown in Table 6.

**Table 7:** Comparison of bitrates in this work and in other PRNGs over ECs.

	Ref.^28^, 2019	Ref.^29^, 2021	This work	
			MATLAB implementaion	C# implementaion
Bitrate in Mbps	0.070444	0.072140	0.09755	0.55869

### Encryption system results

**Figure 10:**
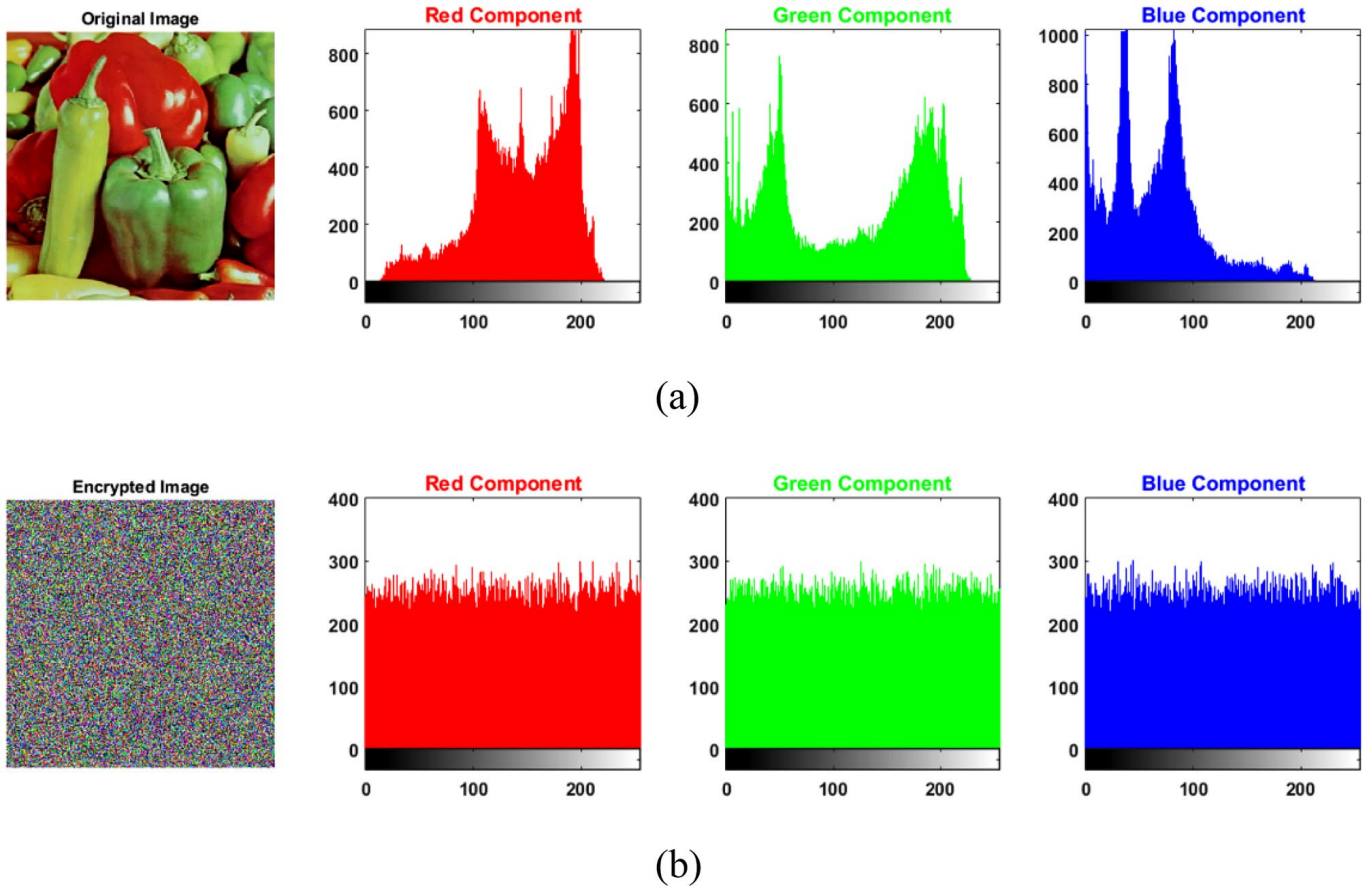
Histograms for the three-color channels in (**a**) Peppers and (**b**) encrypted Peppers.

**Figure 11:**
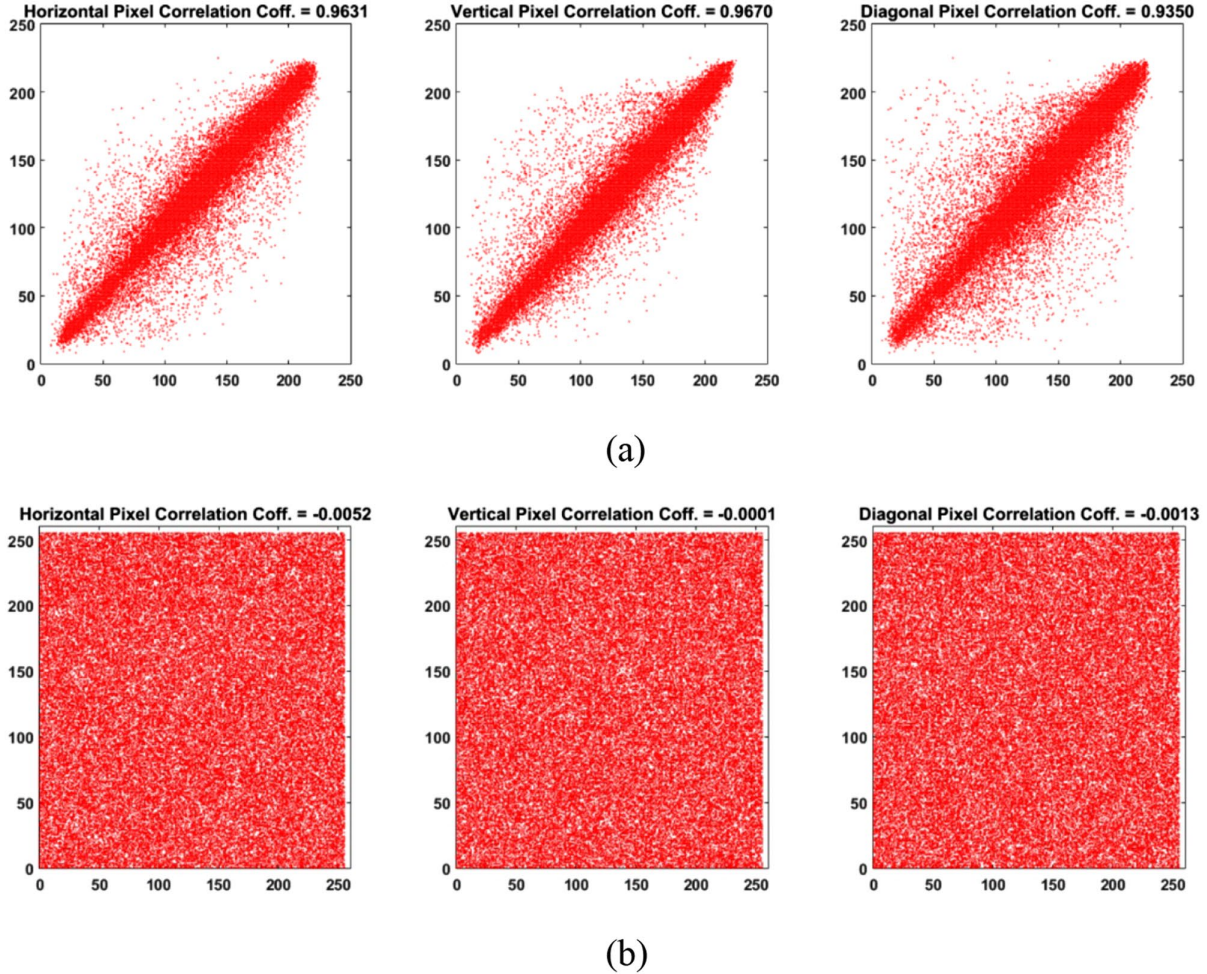
Adjacent pixel values in horizontal, vertical, and diagonal directions in (**a**) Peppers and (**b**) encrypted Peppers for the red channel.

Using the same system key ${K}_{1}$(see Fig. 8), the values for ${a}_{key}$ and ${b}_{key}$ are 222 and 162, respectively. Figure 10 shows the histogram plots for Peppers and encrypted Peppers where the input image has clear peaks while the encrypted image has a uniform distribution across all channels, as supported by the correlation results in Table 8. Furthermore, it is clear from the visual inspection that the encrypted output image shows complete randomness. Figure 11 shows the adjacent pixel values and correlation values in horizontal, vertical, and diagonal directions for the red channel of Peppers and encrypted Peppers. Similar results are achieved in the green and blue channels.

**Table 8:** Analysis results for encrypted Peppers using the system key ${K}_{1}$.

	Pixel correlations			MSE	Entropy	Differential attack measures		
	Horz	Vert	Diag			MAE	NPCR (%)	UACI (%)
R	− 0.0052	− 0.0001	− 0.0013	7703.80	7.9971	72.8052	99.6043	33.4037
G	− 0.0045	0.0008	− 0.0015	11,068.50	7.9973	85.9236	99.6022	33.4538
B	− 0.0028	− 0.0019	0.0001	11,467.20	7.9967	87.5722	99.6123	33.3678
Avg	0.0042	0.0009	0.0010	10,079.84	7.9970	82.1003	99.6063	33.4085

Table 8 shows the correlation results for encrypted Peppers in horizontal, vertical, and diagonal directions. The values are close to zero, indicating how much the pixels are not correlated anymore after the encryption. The differential attack measures are calculated by taking the average values after changing the pixel value in ten random pixels. It is clear from the results that the dependence of Arnold's cat map parameters on the image, as given by Eq. (5), enhanced the results of the differential attack measures. Furthermore, the MSE results show how far is the encrypted image from the source image. At the same time, the entropy values are very close to 8, which provides evidence of the randomness existing in the encrypted images.

**Figure 12:**
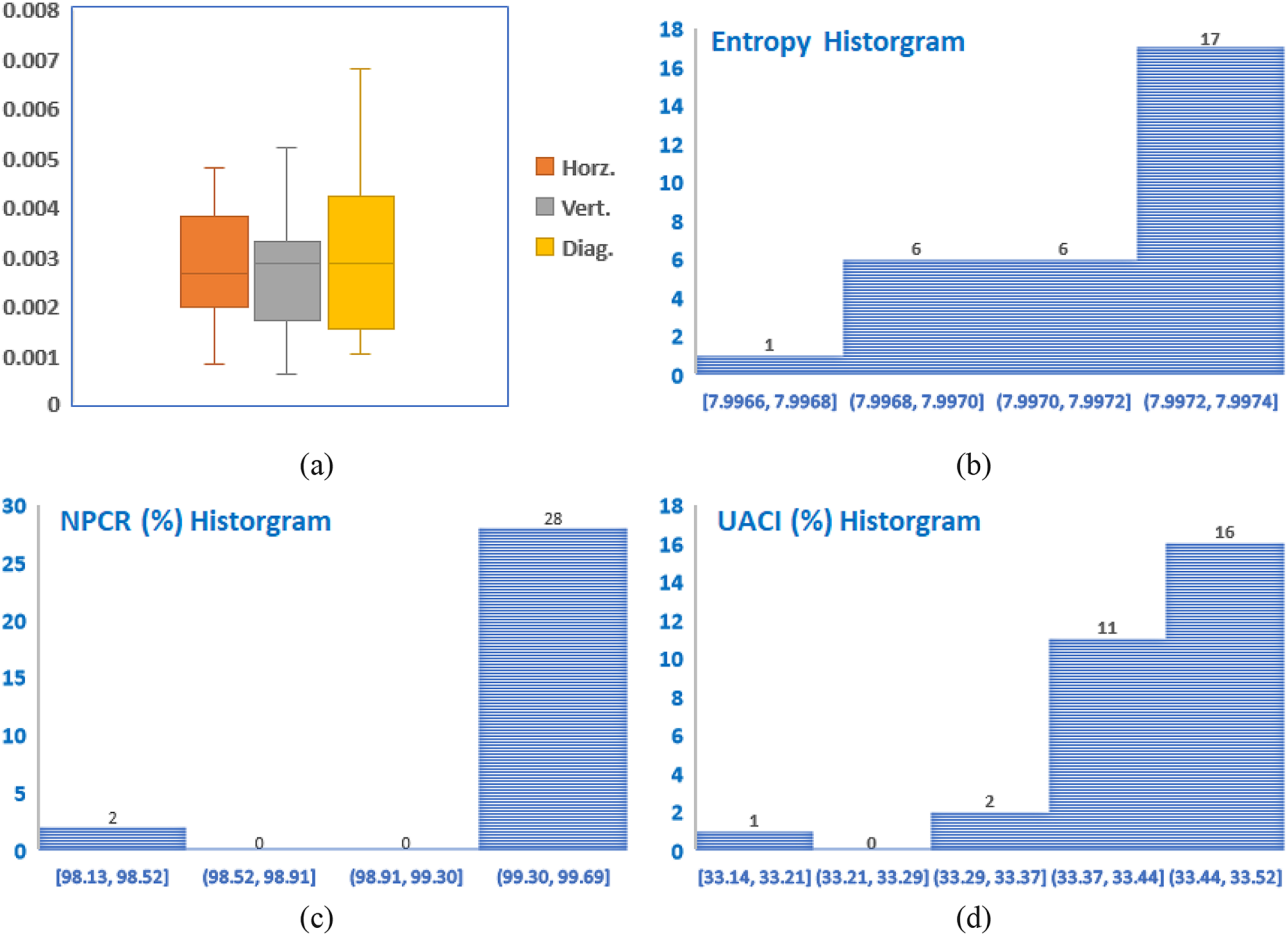
Statistical analysis results for encrypted Peppers using 30 different system keys (${K}_{1}, {K}_{2}, \dots , { K}_{30}$): (**a**) correlation box plot, (**b**) entropy histogram, (**c**) NPCR histogram, and (**d**) UACI histogram.

In addition, Fig. 12 shows the statistical analysis results for encrypted Peppers using 30 different system keys (${K}_{1}, {K}_{2}, \dots , { K}_{30}$). For the box plot, the correlation results in the horizontal, vertical, and diagonal directions are given. The horizontal and vertical results are distributed symmetrically, while the diagonal results are positively skewed. The interquartile maximum range is 0.0027, which means that the three distributions are very concentrated. For the entropy histogram, NPCR histogram, and UACI histogram, it is clear that most of the results fall in the highest range for each test indicating the quality of the encrypted image regardless of the used system key.

Furthermore, Table 9 summarizes the statistical analysis results where all results are in the good, expected ranges. The results provide evidence that the system is stable with respect to different system keys. The small values of the standard deviation demonstrate that, for any system key, the results are expected to be very close to the average results achieved.

**Table 9:** Summary of the statistical analysis results for encrypted Peppers using 30 different system keys (${K}_{1}, {K}_{2}, \dots , { K}_{30}$).

	Pixel correlations			MSE	Entropy	Differential attack measures		
	Horz	Vert	Diag			MAE	NPCR (%)	UACI (%)
Min	0.0008	0.0006	0.0010	9996.03	7.9966	81.6007	98.1324	33.1360
Max	0.0048	0.0069	0.0068	10,132.41	7.9974	82.3008	99.6155	33.5182
Avg	0.0029	0.0028	0.0031	10,055.13	7.9971	81.9509	99.5061	33.4360
Std	0.0011	0.0014	0.0015	28.91	0.0002	0.1504	0.3502	0.0731

Moreover, Table 10 shows the analysis results for different standard images from the USC-SIPI image database^39^ of size $512\times 512$ and the black image. The results show that the system successfully encrypts all images giving good measure values within the required ranges.

**Table 10:** Analysis results for some images from the USC-SIPI image database and the black image.

Img	Original image corr			Encrypted image corr			Entropy		Differential attack measures		
	Horz	Vert	Diag	Horz	Vert	Diag	Orig	Enc	MAE	NPCR (%)	UACI (%)
House	0.9550	0.9563	0.9190	0.0009	0.0023	0.0007	7.3602	7.9993	78.8235	99.6081	33.4772
San Diego 2.1.02	0.7937	0.7731	0.6973	0.0008	0.0023	0.0023	7.1394	7.9994	75.8555	99.5761	33.4548
Oakland 2.1.04	0.7572	0.7814	0.6810	0.0022	0.0006	0.0017	6.3841	7.9993	72.0607	99.5990	33.4842
Woodland 2.1.06	0.9073	0.8948	0.8429	0.0010	0.0022	0.0012	7.3475	7.9993	72.9742	99.6104	33.4827
Earth 2.1.11	0.9629	0.9680	0.9416	0.0010	0.0026	0.0013	6.9287	7.9993	72.0452	99.6077	33.4328
Splash 4.2.01	0.9858	0.9871	0.9751	0.0008	0.0018	0.0027	6.6530	7.9993	86.6092	99.6126	33.4819
Mandrill 4.2.03	0.8986	0.8373	0.8097	0.0009	0.0018	0.0027	7.6444	7.9992	76.3159	99.6054	33.4402
Airplane 4.2.05	0.9648	0.9533	0.9272	0.0018	0.0016	0.0017	6.5768	7.9993	83.0794	99.6035	33.4515
Boat 4.2.06	0.9661	0.9632	0.9493	0.0018	0.0001	0.0020	7.3896	7.9992	82.2794	99.6117	33.4810
Peppers 4.2.07	0.9704	0.9715	0.9576	0.0017	0.0009	0.0025	7.2978	7.9993	82.1370	99.6011	33.4705
Black Image	1.0000	1.0000	1.0000	0.0017	0.0014	0.0009	0.0000	7.9993	127.4746	99.5825	33.4626

### System key sensitivity results

The sensitivity of the system key is examined by changing one bit in it, then decrypting an image with this wrong key and checking the results. Since the system key value is used in calculating the base point ${P}_{0}$ used by the PRNG, any change in any bit produces a new base point. Hence, the PRNG will not be synchronized with the encrypted image. Two cases are examined, Case I, where the least significant bit is changed, and Case II, where the 9th bit is changed. In Case I, the value of ${a}_{key}$ is changed, whereas the value of ${b}_{key}$ is unchanged. While in Case II, the value of ${a}_{key}$ is kept unchanged, whereas the value of ${b}_{key}$ is changed.

**Figure 13:**
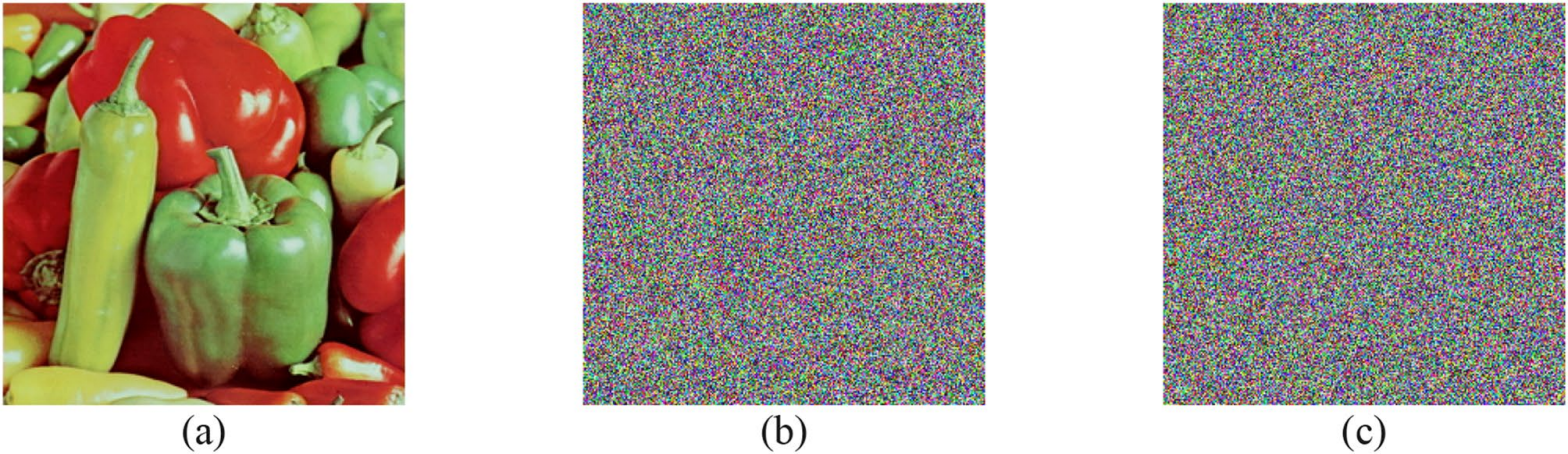
Decryption using (**a**) exact key, (**b**) case I, and (**c**) case II.

Table 11 shows the results for the two test cases. The PRNG was not synchronized with the encrypted image in cases I and II. Therefore, the results for the MSE are large, and entropy values indicate the complete randomness of the wrong decrypted images. These results are supported by visual inspection of the decrypted images, as shown in Fig. 13.

**Table 11:** Decryption results with different keys.

Test	MSE			Entropy		
	Red	Green	Blue	Red	Green	Blue
Exact key	0.00	0.00	0.00	7.2946	7.5483	7.0823
Case I	7732.79	10,904.86	11,449.54	7.9975	7.9974	7.9974
Case II	7694.79	11,013.62	11,400.54	7.9973	7.9979	7.9972

### Computation complexity

The system’s time complexity can be derived by using the system block diagram of Fig. 7. Assuming that the image size is equal to $\mathrm{M}\times \mathrm{N}$, then the PRNG takes $(\mathrm{M}\times \mathrm{N})/8$ iterations to produce the required random numbers. Therefore, the complexity for the PRNG is $\mathcal{O}((\mathrm{M}\times \mathrm{N})/8)\approx \mathcal{O}(\mathrm{M}\times \mathrm{N})$. The substitution stage performs XOR operations for each pixel in the image and, hence, the complexity for the substitution stage is $\mathcal{O}(\mathrm{M}\times \mathrm{N})$. Next, the summation block cumulatively adds all pixel values and, hence, the complexity of this block is $\mathcal{O}(\mathrm{M}\times \mathrm{N})$. Finally, the permutation stage maps each pixel location to a new location and the complexity for this stage is also $\mathcal{O}(\mathrm{M}\times \mathrm{N})$. Therefore, the total complexity for the system is $\mathcal{O}(\mathrm{M}\times \mathrm{N}) + \mathcal{O}(\mathrm{M}\times \mathrm{N}) + \mathcal{O}(\mathrm{M}\times \mathrm{N}) + \mathcal{O}(\mathrm{M}\times \mathrm{N}) = 4\times \mathcal{O}(\mathrm{M}\times \mathrm{N}) \approx \mathcal{O}(\mathrm{M}\times \mathrm{N})$.

### Comparison with related literature

Table 12 compares the results accomplished by this work with other related work in terms of pixel correlations, differential attack measures, and entropy of an encrypted grayscale image of size $256\times 256$. The results show that the security measures are close to each other. Furthermore, Table 13 gives the total execution time, using MATLAB R2015a, for the proposed encryption and decryption systems compared to other related work. The proposed system performance is clearly better.

**Table 12:** Comparison with related work for an image of size $256\times 256$.

Ref. no.	Pixel correlations			Differential attack measures		Entropy
	Horz	Vert	Diag	NPCR (%)	UACI (%)	
Ref.^23^, 2015	0.0025	0.0037	0.0011	99.63	33.56	7.9968
Ref.^28^, 2019	0.0012	0.0003	0.0010	99.60	33.48	7.9993
Ref.^31^, 2021	− 0.0044	− 0.0007	− 0.0031	99.60	33.34	7.9971
This work	0.0027	− 0.00004	− 0.0056	99.59	33.44	7.9971

**Table 13:** Comparing the execution times for an image of size $256\times 256$.

Ref. no.	Encryption + decryption time (s)
Ref.^32^, 2022	21.27
Ref.^41^, 2015	7.73
This work	3.78

## Conclusions

The presented PRNG has a simple and efficient design, which was achieved by utilizing the proposed framework through minimizing the EC and non-EC operations. Consequently, the introduced encryption system utilizes low computational resources and, hence, it is a good candidate for real-time applications.

In conclusion, ECs are good candidates for designing PRNGs. The number of bits in each point coordinate is suitable for bit extraction in secure curves with large prime numbers. Furthermore, the system's security is inherited from the difficulty of the DLP. Finally, the proposed framework for designing PRNGs can help in optimizing the system design by simplifying each block as much as possible, resulting in fast and secure bitstream output. Future work includes enhancing bit extraction criteria to increase the number of bits extracted from each point coordinate and utilizing ECs in generating dynamic S-boxes.

## Supplementary Information

Supplementary Information 1.

Supplementary Information 2.

Supplementary Information 3.

## Data Availability

The data used in this paper are available from the corresponding author upon request.
