# Supplementary material for: A generalized framework for elliptic curves based PRNG and its utilization in image encryption
Source: Sci Rep. 2022 Aug 2;12:13278. doi: 10.1038/s41598-022-17045-x (PMC9346143; doi:10.1038/s41598-022-17045-x)

NIST Report Results for 30 random K values

$K_1 = 337809543519903594669302432357077515672$

Test	PV	PP
Frequency	0.637119	1.000
Block Frequency	0.637119	1.000
Cumulative Sums	0.534146	1.000
Runs	0.012650	1.000
Longest Run	0.213309	1.000
Rank	0.350485	0.958
FFT	0.534146	1.000
Non-Overlapping Template	0.319639	0.990
Overlapping Template	0.275709	0.958
Universal	0.911413	1.000
Approximate Entropy	0.090936	1.000
Random Excursions	0.087587	0.993
Random Excursions Variant	0.066330	0.964
Serial	0.451262	0.979
Linear Complexity	0.275709	1.000
Final Result	Success	

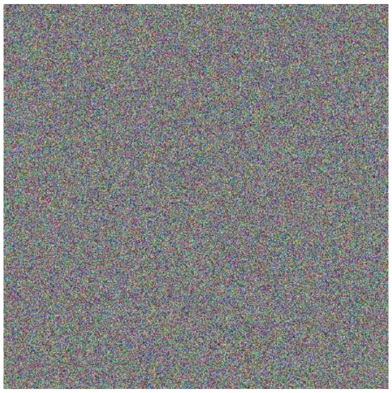

$K_2 = 242944764197277766621257868618941575723$

Test	PV	PP
Frequency	0.437274	1.000
Block Frequency	0.437274	1.000
Cumulative Sums	0.507814	1.000
Runs	0.437274	1.000
Longest Run	0.122325	1.000
Rank	0.122325	1.000
FFT	0.122325	1.000
Non-Overlapping Template	0.344863	0.991
Overlapping Template	0.012650	0.958
Universal	0.437274	0.958
Approximate Entropy	0.637119	1.000
Random Excursions	0.311355	0.981
Random Excursions Variant	0.401450	0.987
Serial	0.208684	1.000
Linear Complexity	0.834308	0.958
Final Result	Success	

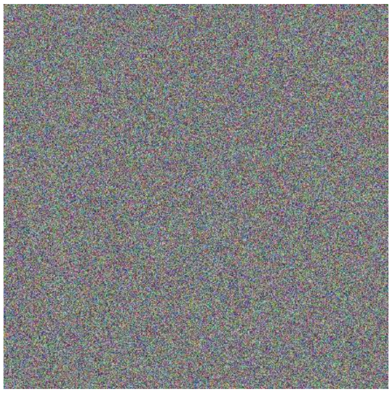

$K_3 = 2357863793657383423643772024584962590 29$

Test	PV	PP
Frequency	0.025193	1.000
Block Frequency	0.437274	0.958
Cumulative Sums	0.630949	1.000
Runs	0.834308	0.958
Longest Run	0.637119	1.000
Rank	0.162606	1.000
FFT	0.534146	1.000
Non-Overlapping Template	0.293808	0.987
Overlapping Template	0.534146	1.000
Universal	0.275709	1.000
Approximate Entropy	0.991468	1.000
Random Excursions	0.239373	0.990
Random Excursions Variant	0.271920	0.996
Serial	0.087628	0.979
Linear Complexity	0.637119	1.000
Final Result	Success	

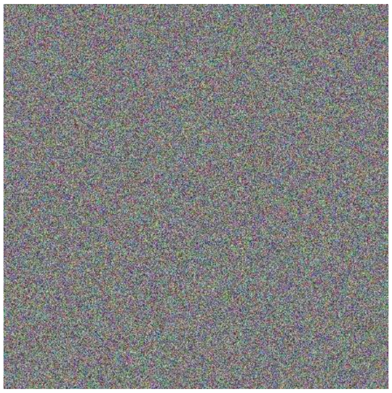

$K_4 = 27743457011761601518595828290930576393 2$

Test	PV	PP
Frequency	0.122325	1.000
Block Frequency	0.035174	1.000
Cumulative Sums	0.462622	0.958
Runs	0.739918	1.000
Longest Run	0.911413	1.000
Rank	0.090936	0.958
FFT	0.739918	0.958
Non-Overlapping Template	0.353159	0.991
Overlapping Template	0.275709	1.000
Universal	0.911413	0.958
Approximate Entropy	0.637119	0.917
Random Excursions	0.257508	0.990
Random Excursions Variant	0.225674	1.000
Serial	0.442316	0.958
Linear Complexity	0.162606	1.000
Final Result	Success	

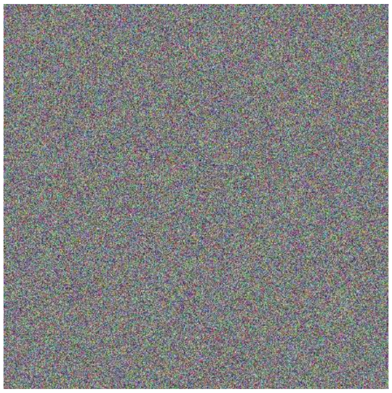

$K_5 = 334184713102307983152604308924564809388$

Test	PV	PP
Frequency	0.834308	1.000
Block Frequency	0.350485	1.000
Cumulative Sums	0.493802	1.000
Runs	0.122325	0.958
Longest Run	0.964295	1.000
Rank	0.048716	1.000
FFT	0.637119	0.958
Non-Overlapping Template	0.291224	0.989
Overlapping Template	0.090936	1.000
Universal	0.090936	1.000
Approximate Entropy	0.025193	1.000
Random Excursions	0.063810	0.977
Random Excursions Variant	0.114510	0.990
Serial	0.242995	1.000
Linear Complexity	0.066882	1.000
Final Result	Success	

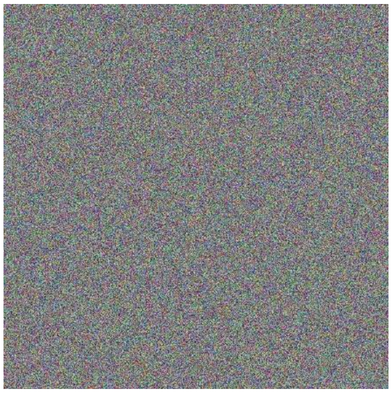

$K_6 = 290173676703331164685891326254904127715$

Test	PV	PP
Frequency	0.834308	1.000
Block Frequency	0.350485	1.000
Cumulative Sums	0.493802	1.000
Runs	0.122325	0.958
Longest Run	0.964295	1.000
Rank	0.048716	1.000
FFT	0.637119	0.958
Non-Overlapping Template	0.291224	0.989
Overlapping Template	0.090936	1.000
Universal	0.090936	1.000
Approximate Entropy	0.025193	1.000
Random Excursions	0.063810	0.977
Random Excursions Variant	0.114510	0.990
Serial	0.242995	1.000
Linear Complexity	0.066882	1.000
Final Result	Success	

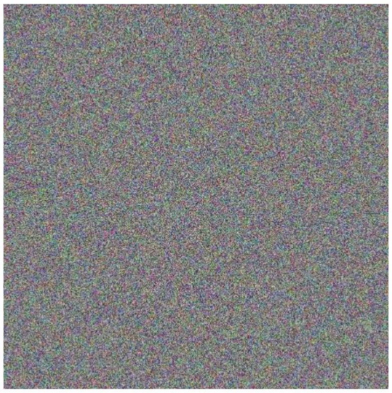

$$K_7 = 226373422207292726520866955115700273892$$

Test	PV	PP
Frequency	0.350485	1.000
Block Frequency	0.637119	1.000
Cumulative Sums	0.684227	1.000
Runs	0.017912	1.000
Longest Run	0.534146	0.958
Rank	0.122325	1.000
FFT	0.437274	1.000
Non-Overlapping Template	0.317897	0.991
Overlapping Template	0.739918	1.000
Universal	0.162606	0.958
Approximate Entropy	0.350485	1.000
Random Excursions	0.291009	0.990
Random Excursions Variant	0.267003	1.000
Serial	0.155442	0.979
Linear Complexity	0.637119	1.000
Final Result	Success	

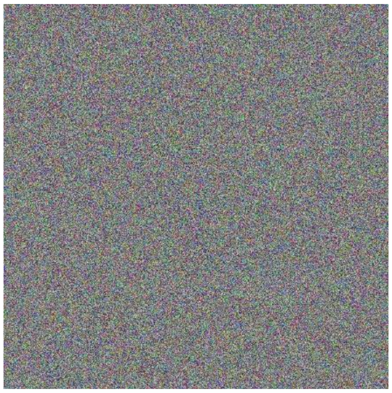

$$K_8 = 272007293667070671861552978337785766085$$

Test	PV	PP
Frequency	0.066882	1.000
Block Frequency	0.834308	1.000
Cumulative Sums	0.066882	1.000
Runs	0.122325	1.000
Longest Run	0.162606	0.958
Rank	0.437274	1.000
FFT	0.066882	1.000
Non-Overlapping Template	0.373447	0.989
Overlapping Template	0.911413	1.000
Universal	0.213309	1.000
Approximate Entropy	0.437274	0.958
Random Excursions	0.064022	1.000
Random Excursions Variant	0.082524	0.993
Serial	0.373728	1.000
Linear Complexity	0.275709	1.000
Final Result	Success	

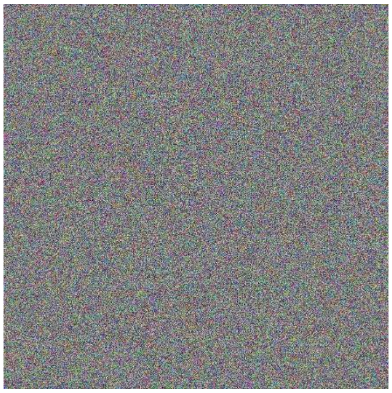

$K_9 =$ **230382529633454484299113379702857890383**

Test	PV	PP
Frequency	0.162606	1.000
Block Frequency	0.534146	1.000
Cumulative Sums	0.098890	0.979
Runs	0.025193	0.958
Longest Run	0.911413	1.000
Rank	0.637119	1.000
FFT	0.739918	1.000
Non-Overlapping Template	0.369002	0.991
Overlapping Template	0.035174	1.000
Universal	0.637119	1.000
Approximate Entropy	0.035174	1.000
Random Excursions	0.027317	0.993
Random Excursions Variant	0.038812	0.991
Serial	0.224962	0.979
Linear Complexity	0.739918	1.000
Final Result	Success	

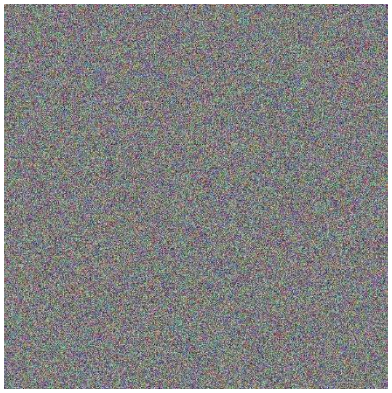

$K_{10} =$ **281207271589818928219323850943056256420**

Test	PV	PP
Frequency	0.275709	1.000
Block Frequency	0.275709	0.958
Cumulative Sums	0.252078	1.000
Runs	0.213309	1.000
Longest Run	0.162606	1.000
Rank	0.437274	1.000
FFT	0.213309	1.000
Non-Overlapping Template	0.365356	0.988
Overlapping Template	0.066882	1.000
Universal	0.213309	1.000
Approximate Entropy	0.275709	1.000
Random Excursions	0.215784	0.967
Random Excursions Variant	0.231672	1.000
Serial	0.231234	1.000
Linear Complexity	0.834308	0.958
Final Result	Success	

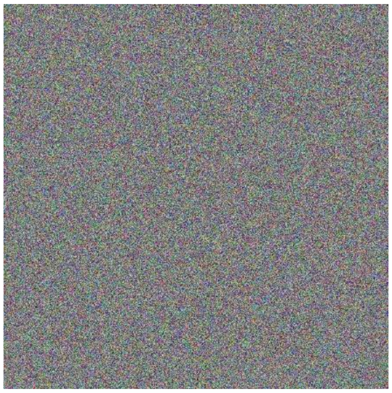

$K_{11} = 32203768430655436990058909751430144418 8$

Test	PV	PP
Frequency	0.911413	0.917
Block Frequency	0.437274	0.958
Cumulative Sums	0.281897	0.917
Runs	0.350485	1.000
Longest Run	0.350485	1.000
Rank	0.090936	1.000
FFT	0.035174	0.958
Non-Overlapping Template	0.337603	0.988
Overlapping Template	0.637119	1.000
Universal	0.012650	1.000
Approximate Entropy	0.437274	1.000
Random Excursions	0.236432	1.000
Random Excursions Variant	0.234605	1.000
Serial	0.033442	0.979
Linear Complexity	0.213309	1.000
Final Result	Success	

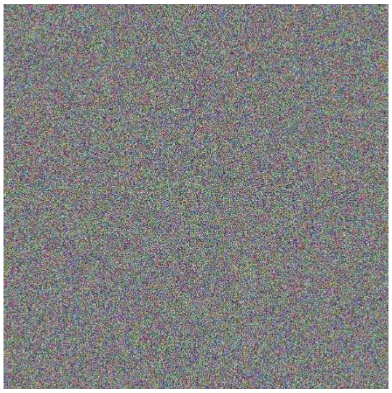

$K_{12} = 33203527490388734384765533216678131701 1$

Test	PV	PP
Frequency	0.162606	1.000
Block Frequency	0.834308	1.000
Cumulative Sums	0.637032	0.979
Runs	0.834308	0.958
Longest Run	0.162606	1.000
Rank	0.025193	0.958
FFT	0.122325	1.000
Non-Overlapping Template	0.344261	0.991
Overlapping Template	0.739918	1.000
Universal	0.911413	1.000
Approximate Entropy	0.213309	0.958
Random Excursions	0.353196	0.971
Random Excursions Variant	0.239219	1.000
Serial	0.431122	1.000
Linear Complexity	0.437274	0.958
Final Result	Success	

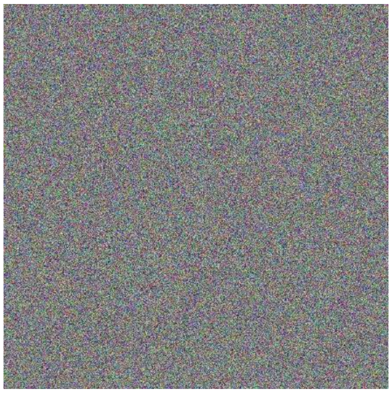

$K_{13} = 197812599167446168037275290663313107652$

Test	PV	PP
Frequency	0.911413	1.000
Block Frequency	0.162606	1.000
Cumulative Sums	0.545202	0.979
Runs	0.017912	1.000
Longest Run	0.213309	1.000
Rank	0.162606	0.917
FFT	0.035174	0.958
Non-Overlapping Template	0.338509	0.991
Overlapping Template	0.834308	1.000
Universal	0.739918	1.000
Approximate Entropy	0.008879	1.000
Random Excursions	0.182274	0.977
Random Excursions Variant	0.089156	0.990
Serial	0.007347	1.000
Linear Complexity	0.437274	1.000
Final Result	Success	

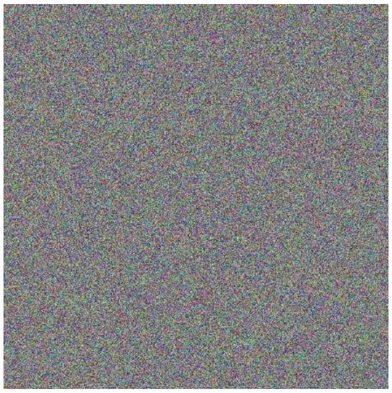

$K_{14} = 313459482441357487056000709615786342877$

Test	PV	PP
Frequency	0.122325	1.000
Block Frequency	0.534146	0.958
Cumulative Sums	0.276029	1.000
Runs	0.162606	1.000
Longest Run	0.275709	1.000
Rank	0.350485	1.000
FFT	0.437274	0.917
Non-Overlapping Template	0.359707	0.993
Overlapping Template	0.090936	0.958
Universal	0.213309	1.000
Approximate Entropy	0.162606	0.958
Random Excursions	0.032630	0.993
Random Excursions Variant	0.036374	0.994
Serial	0.485710	0.979
Linear Complexity	0.048716	1.000
Final Result	Success	

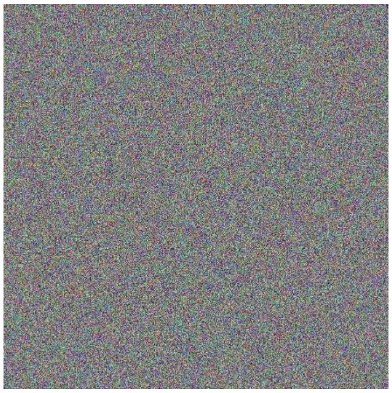

$K_{15} = 272399429113698398355723801540004329391$

Test	PV	PP
Frequency	0.739918	0.958
Block Frequency	0.162606	1.000
Cumulative Sums	0.537197	0.958
Runs	0.637119	0.958
Longest Run	0.350485	1.000
Rank	0.911413	1.000
FFT	0.035174	0.958
Non-Overlapping Template	0.347766	0.991
Overlapping Template	0.012650	1.000
Universal	0.008879	0.958
Approximate Entropy	0.637119	1.000
Random Excursions	0.124439	0.992
Random Excursions Variant	0.091758	0.993
Serial	0.688519	1.000
Linear Complexity	0.122325	1.000
Final Result	Success	

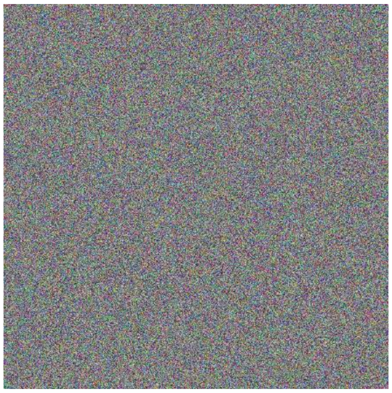

$K_{16} = 236299200458710537629694834190626673028$

Test	PV	PP
Frequency	0.122325	1.000
Block Frequency	0.834308	1.000
Cumulative Sums	0.348376	1.000
Runs	0.637119	1.000
Longest Run	0.911413	0.958
Rank	0.275709	1.000
FFT	0.437274	1.000
Non-Overlapping Template	0.325018	0.989
Overlapping Template	0.637119	1.000
Universal	0.275709	1.000
Approximate Entropy	0.437274	1.000
Random Excursions	0.410123	1.000
Random Excursions Variant	0.311517	1.000
Serial	0.213309	1.000
Linear Complexity	0.534146	1.000
Final Result	Success	

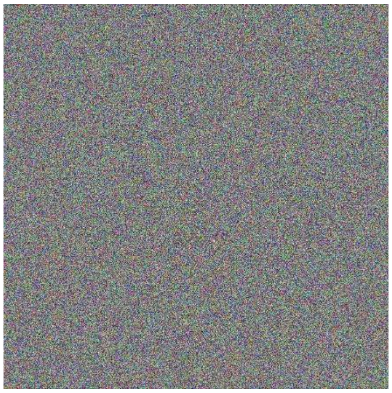

$K_{17} = 206622649562577773750641098071011677260$

Test	PV	PP
Frequency	0.122325	1.000
Block Frequency	0.637119	0.958
Cumulative Sums	0.012054	1.000
Runs	0.275709	0.958
Longest Run	0.162606	1.000
Rank	0.090936	1.000
FFT	0.213309	1.000
Non-Overlapping Template	0.345230	0.993
Overlapping Template	0.275709	1.000
Universal	0.122325	0.958
Approximate Entropy	0.437274	0.958
Random Excursions	0.107324	1.000
Random Excursions Variant	0.108451	0.997
Serial	0.187958	0.958
Linear Complexity	0.090936	0.958
Final Result	Success	

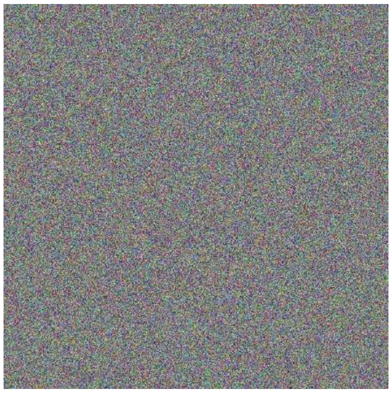

$K_{18} = 287071537296860748663400559723285735362$

Test	PV	PP
Frequency	0.275709	1.000
Block Frequency	0.122325	1.000
Cumulative Sums	0.476614	1.000
Runs	0.437274	1.000
Longest Run	0.437274	1.000
Rank	0.911413	1.000
FFT	0.006196	1.000
Non-Overlapping Template	0.328041	0.986
Overlapping Template	0.162606	1.000
Universal	0.437274	1.000
Approximate Entropy	0.964295	0.958
Random Excursions	0.148178	1.000
Random Excursions Variant	0.220699	1.000
Serial	0.300514	1.000
Linear Complexity	0.162606	1.000
Final Result	Success	

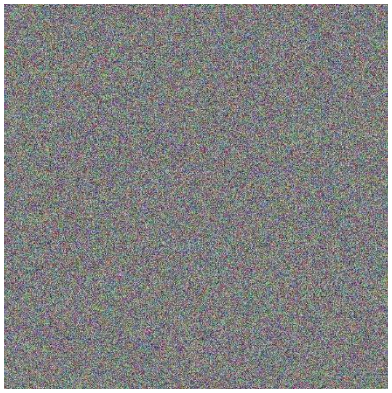

$K_{19} = 204715239061880653387862189332361403524$

Test	PV	PP
Frequency	0.739918	1.000
Block Frequency	0.534146	0.958
Cumulative Sums	0.152123	1.000
Runs	0.275709	0.958
Longest Run	0.350485	1.000
Rank	0.275709	1.000
FFT	0.090936	1.000
Non-Overlapping Template	0.309144	0.993
Overlapping Template	0.739918	1.000
Universal	0.437274	1.000
Approximate Entropy	0.437274	1.000
Random Excursions	0.122901	0.969
Random Excursions Variant	0.087242	0.969
Serial	0.299940	1.000
Linear Complexity	0.090936	0.958
Final Result	Success	

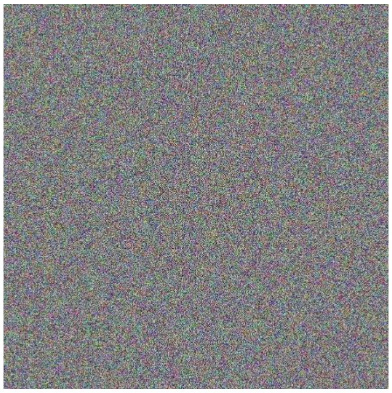

$K_{20} = 301584539535497696648130288290944605892$

Test	PV	PP
Frequency	0.122325	1.000
Block Frequency	0.090936	1.000
Cumulative Sums	0.019738	1.000
Runs	0.964295	1.000
Longest Run	0.637119	1.000
Rank	0.035174	0.958
FFT	0.834308	1.000
Non-Overlapping Template	0.341598	0.988
Overlapping Template	0.275709	0.958
Universal	0.012650	1.000
Approximate Entropy	0.437274	1.000
Random Excursions	0.230065	0.992
Random Excursions Variant	0.170598	1.000
Serial	0.085521	0.958
Linear Complexity	0.213309	1.000
Final Result	Success	

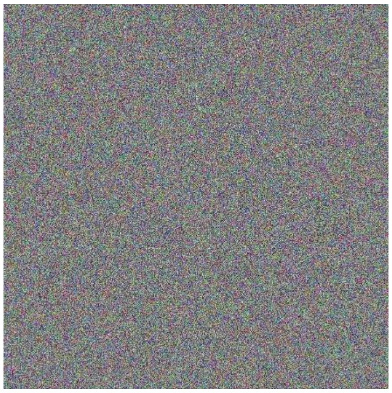

$K_{21} = 293585794790628437062416399539719212463$

Test	PV	PP
Frequency	0.637119	0.958
Block Frequency	0.834308	0.917
Cumulative Sums	0.592397	0.979
Runs	0.275709	0.958
Longest Run	0.122325	1.000
Rank	0.162606	1.000
FFT	0.534146	1.000
Non-Overlapping Template	0.322275	0.991
Overlapping Template	0.350485	1.000
Universal	0.213309	1.000
Approximate Entropy	0.012650	1.000
Random Excursions	0.149992	0.982
Random Excursions Variant	0.172684	0.972
Serial	0.069826	1.000
Linear Complexity	0.437274	1.000
Final Result	Success	

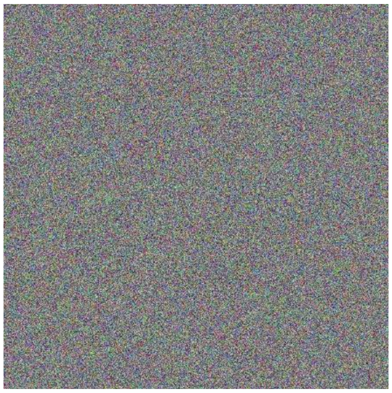

$K_{22} = 339074228441080644951372042095769275528$

Test	PV	PP
Frequency	0.637119	1.000
Block Frequency	0.637119	0.958
Cumulative Sums	0.352001	0.979
Runs	0.637119	1.000
Longest Run	0.025193	0.958
Rank	0.350485	0.958
FFT	0.162606	1.000
Non-Overlapping Template	0.333072	0.990
Overlapping Template	0.534146	0.958
Universal	0.122325	1.000
Approximate Entropy	0.162606	1.000
Random Excursions	0.019537	1.000
Random Excursions Variant	0.030978	0.988
Serial	0.183323	1.000
Linear Complexity	0.090936	1.000
Final Result	Success	

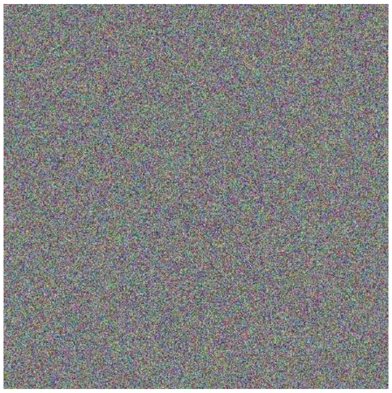

$$K_{23} = 278106158596303413681761330899706309252$$

Test	PV	PP
Frequency	0.437274	1.000
Block Frequency	0.275709	1.000
Cumulative Sums	0.106631	1.000
Runs	0.162606	0.958
Longest Run	0.437274	1.000
Rank	0.162606	1.000
FFT	0.275709	1.000
Non-Overlapping Template	0.360528	0.994
Overlapping Template	0.637119	1.000
Universal	0.213309	1.000
Approximate Entropy	0.213309	1.000
Random Excursions	0.102436	0.977
Random Excursions Variant	0.179874	1.000
Serial	0.069826	0.979
Linear Complexity	0.066882	1.000
Final Result	Success	

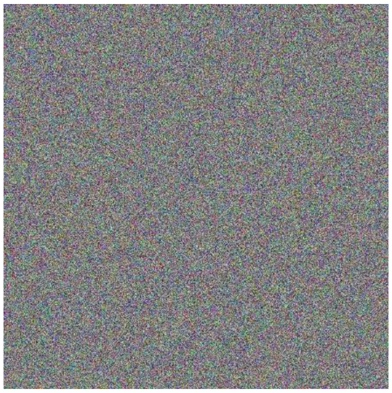

$$K_{24} = 206218733527383749929152780800883058953$$

Test	PV	PP
Frequency	0.066882	0.958
Block Frequency	0.122325	1.000
Cumulative Sums	0.404928	0.958
Runs	0.213309	1.000
Longest Run	0.911413	1.000
Rank	0.275709	0.958
FFT	0.025193	1.000
Non-Overlapping Template	0.351810	0.989
Overlapping Template	0.637119	1.000
Universal	0.213309	1.000
Approximate Entropy	0.739918	1.000
Random Excursions	0.049976	1.000
Random Excursions Variant	0.149605	0.997
Serial	0.364028	1.000
Linear Complexity	0.025193	1.000
Final Result	Success	

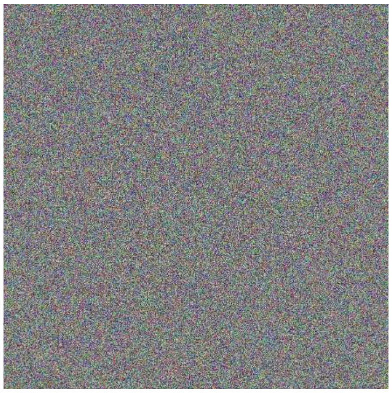

$K_{25} = 321338825084792460872942494297318903286$

Test	PV	PP
Frequency	0.534146	0.958
Block Frequency	0.534146	1.000
Cumulative Sums	0.700785	0.958
Runs	0.534146	1.000
Longest Run	0.350485	1.000
Rank	0.025193	1.000
FFT	0.017912	0.958
Non-Overlapping Template	0.401030	0.994
Overlapping Template	0.350485	1.000
Universal	0.911413	1.000
Approximate Entropy	0.534146	1.000
Random Excursions	0.396886	1.000
Random Excursions Variant	0.423463	0.995
Serial	0.024578	1.000
Linear Complexity	0.275709	1.000
Final Result	Success	

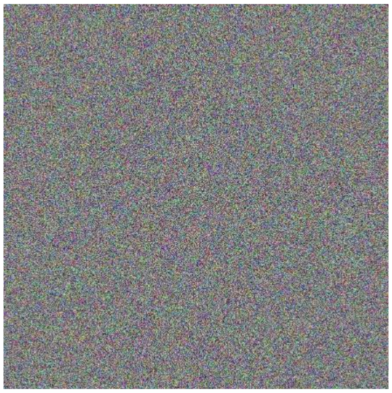

$K_{26} = 194826809547022550307171411742486156253$

Test	PV	PP
Frequency	0.637119	1.000
Block Frequency	0.739918	1.000
Cumulative Sums	0.585633	1.000
Runs	0.350485	0.917
Longest Run	0.350485	1.000
Rank	0.739918	1.000
FFT	0.090936	1.000
Non-Overlapping Template	0.365059	0.987
Overlapping Template	0.122325	1.000
Universal	0.275709	0.958
Approximate Entropy	0.213309	1.000
Random Excursions	0.054814	1.000
Random Excursions Variant	0.023056	1.000
Serial	0.545202	0.979
Linear Complexity	0.739918	1.000
Final Result	Success	

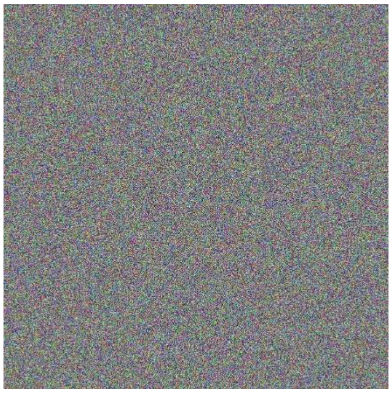

$K_{27} = 265687430827195128623670433118196280456$

Test	PV	PP
Frequency	0.048716	1.000
Block Frequency	0.637119	1.000
Cumulative Sums	0.067488	1.000
Runs	0.122325	1.000
Longest Run	0.437274	1.000
Rank	0.275709	1.000
FFT	0.275709	1.000
Non-Overlapping Template	0.376536	0.993
Overlapping Template	0.048716	1.000
Universal	0.275709	0.958
Approximate Entropy	0.001399	1.000
Random Excursions	0.048622	1.000
Random Excursions Variant	0.073118	0.980
Serial	0.735714	1.000
Linear Complexity	0.437274	0.958
Final Result	Success	

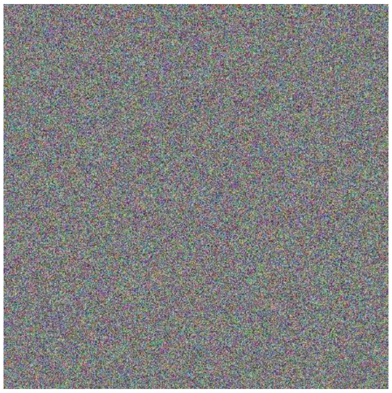

$K_{28} = 229340732209894698747121052368518423911$

Test	PV	PP
Frequency	0.275709	1.000
Block Frequency	0.090936	0.958
Cumulative Sums	0.244509	1.000
Runs	0.090936	1.000
Longest Run	0.275709	0.958
Rank	0.008879	1.000
FFT	0.122325	1.000
Non-Overlapping Template	0.383352	0.988
Overlapping Template	0.122325	0.958
Universal	0.017912	1.000
Approximate Entropy	0.275709	0.958
Random Excursions	0.081704	1.000
Random Excursions Variant	0.135069	1.000
Serial	0.279800	1.000
Linear Complexity	0.012650	1.000
Final Result	Success	

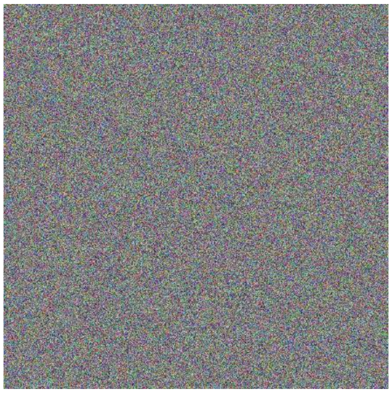

$$K_{29} = 287058049390465765899824682494100273683$$

Test	PV	PP
Frequency	0.350485	1.000
Block Frequency	0.162606	1.000
Cumulative Sums	0.064261	1.000
Runs	0.834308	1.000
Longest Run	0.275709	0.958
Rank	0.437274	1.000
FFT	0.437274	1.000
Non-Overlapping Template	0.327610	0.988
Overlapping Template	0.066882	1.000
Universal	0.350485	0.958
Approximate Entropy	0.035174	1.000
Random Excursions	0.112313	1.000
Random Excursions Variant	0.171097	1.000
Serial	0.325292	0.979
Linear Complexity	0.637119	1.000
Final Result	Success	

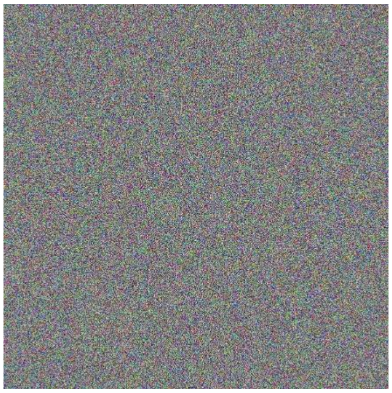

$$K_{30} = 237081826857317528968122432232804860063$$

Test	PV	PP
Frequency	0.437274	1.000
Block Frequency	0.437274	1.000
Cumulative Sums	0.478317	1.000
Runs	0.739918	1.000
Longest Run	0.035174	1.000
Rank	0.350485	1.000
FFT	0.739918	1.000
Non-Overlapping Template	0.311064	0.992
Overlapping Template	0.534146	0.917
Universal	0.017912	1.000
Approximate Entropy	0.066882	0.958
Random Excursions	0.267256	1.000
Random Excursions Variant	0.266158	1.000
Serial	0.404928	1.000
Linear Complexity	0.739918	1.000
Final Result	Success	

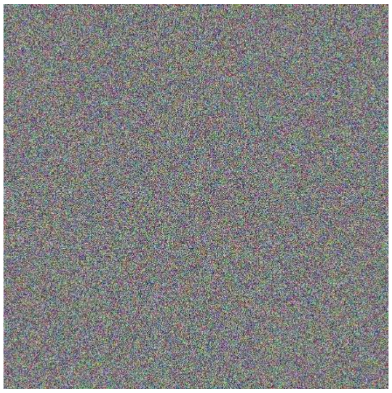

Supplement: Supplementary file 1 — Supplementary Information 1. [file 41598_2022_17045_MOESM1_ESM.pdf]
