# Supplementary material for: A generalized framework for elliptic curves based PRNG and its utilization in image encryption
Source: Sci Rep. 2022 Aug 2;12:13278. doi: 10.1038/s41598-022-17045-x (PMC9346143; doi:10.1038/s41598-022-17045-x)
Supplement: Supplementary file 2 — Supplementary Information 2. [file 41598_2022_17045_MOESM2_ESM.docx]

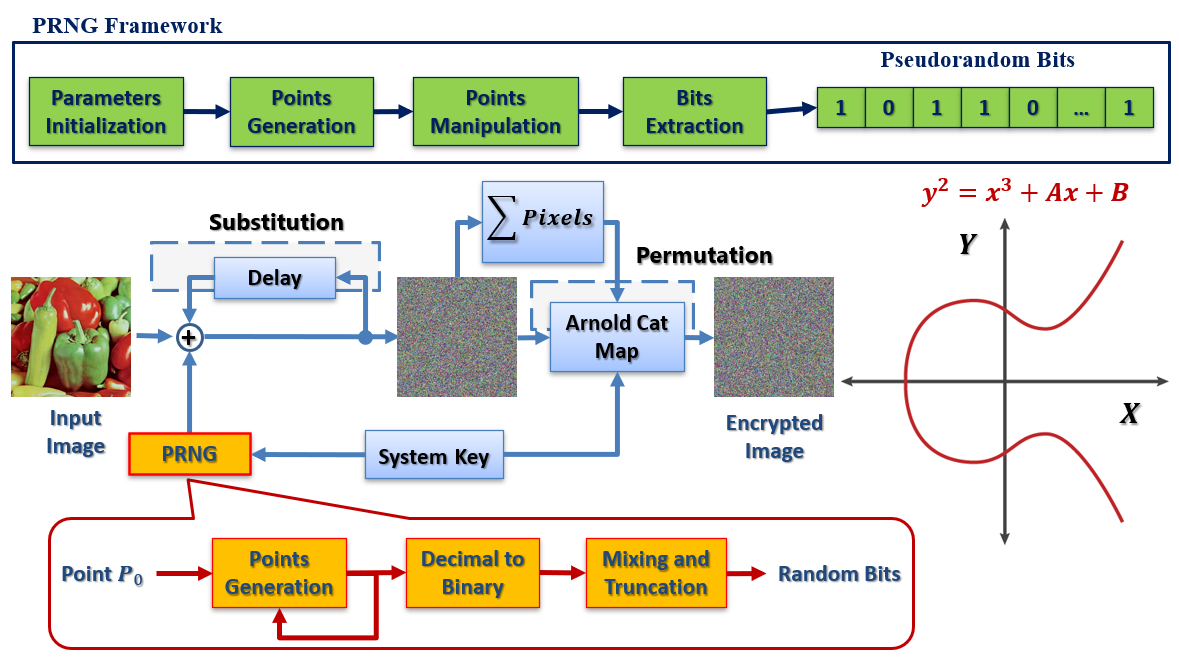


Supplementary figure 1: The block diagram for the proposed generalized framework for elliptic curves based PRNG is at the top. A generic elliptic curve is on the right. The block diagram for the proposed encryption system is in the middle, and at the bottom is the block diagram for the proposed PRNG.
